# Applying system support mapping combined with the matrixed multiple case study approach to identify and analyse implementation determinants: lessons from a multi-site implementation study in German neonatal intensive care units

**DOI:** 10.1186/s12913-026-15507-w

**Published:** 2026-09-05

**Authors:** Nicola Gabriela Dymek, Julia Jaschke, Sarah Meyer, Anna Katharina Stirner, Daniel Wiesen, Till Dresbach, Nadine Scholten, Juliane Köberlein-Neu

**Affiliations:** 1 Centre for Health Economics and Health Services Research, University of Wuppertal, Wuppertal, Germany; 2 Department of Business Administration and Health Care Management, University of Cologne, Cologne, Germany; 3 Erasmus School of Health Policy & Management (ESHPM), Erasmus University Rotterdam, Rotterdam, Netherlands; 4 University Hospital Bonn, Bonn, Germany; 5 Institute of Medical Sociology, Health Services Research and Rehabilitation Science, Faculty of Medicine and University Hospital Cologne, University of Cologne, Cologne, Germany; 6 Center for Health Communication and Health Services Research, Department for Psychosomatic Medicine and Psychotherapy, University Hospital Bonn, Bonn, Germany

**Keywords:** System support mapping, Matrixed multiple case study, Implementation determinants, Context assessment, Implementation research methods, Multi-site implementation, Neonatal intensive care unit

## Abstract

**Background:**

Identifying and understanding implementation determinants is a critical step in the process of tailoring implementation strategies to local contexts. System Support Mapping (SSM) is a method rooted in systems thinking that offers a structured approach to exploring the responsibilities, needs, resources, and wishes of individuals within an implementation context. However, SSM has not yet been systematically described and applied as a methodology for identifying implementation determinants within multi-site implementation studies. This article introduces and illustrates the combined application of SSM and the Matrixed Multiple Case Study (MMCS) approach as a structured six-step methodology for identifying and analysing implementation determinants within and across sites, using data from the Neo-MILK project as an illustrative example. The project aimed to implement a structured lactation support programme for mothers of very low birth weight infants and establish human donor milk banks in German neonatal intensive care units (NICUs).

**Methods:**

To illustrate the six-step methodology, semi-structured interviews with key persons from five German NICUs, which were conducted during the early intervention phase, served as the empirical basis for demonstrating each methodological step. Transcripts were analysed using directed content analysis, with SSM elements serving as deductive categories and context-specific codes developed inductively. The MMCS approach was used to organise, compare, and synthesise findings within and across sites.

**Results:**

Applying the six-step methodology, we demonstrate the type of output each step produces, ranging from individual System Support Maps per key person and NICU (steps 1–3) to a sortable cross-site matrix (steps 4–6). The within-site analysis (step 5) illustrates how the methodology surfaces context-dependent perceptions of the same determinant, showing that a given resource category could be perceived as facilitating, inhibiting, or both, depending on individual responsibilities, needs, and local context. The cross-site analysis (step 6) demonstrates how the matrix structure makes homogeneous and heterogeneous determinant patterns visible across sites, indicating different contextual manifestations of the same determinant.

**Conclusion:**

The combined application of SSM and the MMCS approach provides a structured methodology for identifying and analysing implementation determinants that accounts for the perspectives of individual interest-holders and enables systematic within-site and cross-site comparison. The distinction between homogeneous and heterogeneous determinant patterns offers a basis for differentiating between cross-site and site-specific implementation strategies. Further research is needed to evaluate the use of SSM across multiple evaluation timepoints and its integration into initial and ongoing tailoring of implementation strategies.

**Trial registration:**

DRKS00025058; registered 6 May 2021.

**Supplementary Information:**

The online version contains supplementary material available at https://doi.org/10.1186/s12913-026-15507-w.

## Background

The identification and analysis of implementation determinants, defined as the contextual factors that facilitate or inhibit the implementation of evidence-based interventions (EBIs) in practice, constitutes a critical methodological task in implementation research and practice [1–3]. As the selection, design, and adaptation of implementation strategies are contingent upon an accurate and context-sensitive understanding of local barriers and facilitators [1, 3, 4], the quality of this initial assessment may have considerable implications for the effectiveness of the subsequent implementation tailoring, which can be understood as the systematic process of linking contextual factors to appropriate implementation strategies [1, 5–9]. A tailoring process typically extends from the identification and prioritisation of determinants through the selection, operationalisation, and execution of corresponding implementation strategies [10–18] (see Fig. 2 for an overview). The present paper focuses on step 1, that is, on a methodology for identifying and analysing implementation determinants. The structured outputs generated by this methodology may also serve as a basis for subsequent tailoring steps, although this potential application warrants further investigation and is not within the scope of the present paper.

Various methodological approaches have been proposed for the identification of implementation determinants, ranging from brainstorming, interviews and structured group discussion [19] as well as framework-guided interviews [17] to more pragmatic approaches such as the Pragmatic Context Assessment Tool (pCAT), which is based on the Consolidated Framework for Implementation Research (CFIR) [20]. These approaches vary in the extent to which they (a) capture the holistic, interest-holder-centred nature of implementation contexts, (b) enable within-site comparison across different perspectives, and (c) support systematic cross-site synthesis. A methodology that brings together these analytical dimensions may offer particular value in multi-site implementation research, where both depth of context understanding and breadth of cross-site comparability are desirable [21, 22]. The present paper describes and illustrates such a methodology, combining System Support Mapping (SSM), a potentially valuable but underexplored approach in this context, with the Matrixed Multiple Case Study (MMCS) approach.

SSM is rooted in the field of systems thinking and was developed as a practical method to help Maternal and Child Health professionals describe how they approach a particular implementation process and identify specific priorities for change. It addresses the need to systematically explore roles and responsibilities, identify deficiencies and redundancies in efforts and resources within a system, and prioritise system-level improvement targets [23]. The core elements of a System Support Map are an individual’s perceived role(s) and their associated responsibilities, needs, resources, and wishes regarding a specific process or system (for a detailed description of SSM, see Additional file 1; a summary of SSM elements and their definitions is provided in Table 1 in the Methods section). SSM has been applied in various system-strengthening initiatives, including developmental screening and referral, postpartum care, community-academic research partnerships, youth mental health support, integrated physical and behavioural health, pain management in older adults, and family planning [24, 25]. In these applications, SSM has primarily been used as a self-reporting tool for individuals or implementation teams. To date, however, SSM has not been described as an interview-based analytical methodology for systematically identifying and analysing implementation determinants in multi-site implementation research. A key feature of SSM is its holistic perspective. By considering how the responsibilities, needs, resources, and wishes of individuals interrelate, SSM can reveal the mechanisms through which contextual factors facilitate or inhibit implementation, making it a potentially valuable method for early-stage context assessment. While the SSM literature describes approaches to aggregating data across multiple participants within a single implementation context [23, 24], a structured methodology that systematically combines SSM with cross-site comparison in multi-site implementation research has not yet been established.

The MMCS approach [26] offers a particularly systematic structure for organising, comparing, and synthesising data across multiple cases, which extends the analytical scope of SSM. In implementation research, MMCS has been applied to examine how implementation processes unfold across different sites, enabling researchers to identify both common patterns and site-specific variations [27, 28]. The approach uses a matrix structure in which data sources (e.g., interviews) are organised in columns and analytical categories in rows, allowing systematic within-case and cross-case comparison. The combination of SSM and MMCS may therefore address an important methodological need in multi-site implementation research. SSM provides the conceptual framework for capturing holistic, interest-holder-centred data on implementation contexts, while MMCS provides the analytical structure for systematic comparison and synthesis within and across sites. To our knowledge, this combination has not yet been described or applied in the implementation science literature.

In this paper, we aim to describe and illustrate the combined application of SSM and MMCS within the formative evaluation (FE) of the Neo-MILK project, a multi-site effectiveness-implementation study, which intended to develop and implement a structured lactation and breastfeeding support programme and to establish human donor milk banks (HDMBs) in German neonatal intensive care units (NICUs). The implementation of such programmes in clinical settings is typically complex and requires structured approaches to identify and address local implementation barriers and facilitators [1, 9, 29]. Specifically, we aim to (1) describe the combined SSM-MMCS approach as a structured six-step methodology for identifying and analysing implementation determinants within and across implementation sites, (2) illustrate the outputs and methodological insights generated by this approach, using data from the first evaluation loop of the Neo-MILK FE, and (3) reflect on the strengths, challenges, and potential applications of this combined approach for context assessment in implementation research and practice.

## Methods

### Study design and setting

#### Research context

The Neo-MILK project was initiated to improve provision of mother’s own milk (MOM) or, in case of MOM not or not yet being available, human donor milk (HDM) for very low birth weight (VLBW) infants in German level I NICUs (highest neonatal care level [30]. A structured lactation and breastfeeding support programme was developed and implemented, explicitly adapted to the needs of parents of VLBW infants, and HDMBs were established to promote a uniformly standardised distribution of HDM in German NICUs. The development process of the intervention components, which are listed in accordance with the Template for Intervention Description and Replication (TIDieR) [31] in the Supplementary Table 1, is described elsewhere [32]. Initial implementation was facilitated by strategies identified during the intervention development phase, based on evidence from the literature and two mixed methods surveys (qualitative and quantitative). Moreover, a survey was conducted of mothers of VLBW infants, senior NICU physicians/nurses and key persons in already existing HDMBs across Germany [32]. The initial implementation strategies are presented in the Supplementary Table 2 in accordance with the recommendations set forth by Proctor et al. [33].

The implementation was accompanied by a hybrid type 1 effectiveness-implementation cluster-randomised controlled trial (c-RCT) with a stepped wedge design, consisting of a summative evaluation to test the effectiveness of the intervention and a FE including a process evaluation. As per study design, all participating NICUs started with a control phase which was followed by an intervention phase. Randomisation took place at NICU level. The participating level I NICUs (also referred to as “sites” in the following) were randomly assigned to one of three groups (5 NICUs per group), which proceeded from the control phase into the implementation phase at three different time points. The c-RCT was conducted over 26 months. Before conducting the trial, ethical approval was obtained and the study was preregistered (DRKS00025058; German Clinical Trials Register (*Deutsches Register Klinischer Studien*); https://www.bfarm.de/DE/Das-BfArM/Aufgaben/Deutsches-Register-Klinischer-Studien/_node.html; registration date: 6 May 2021). Further trial details are available in the study protocol [34].

The FE, of which SSM was a core component, was designed in three successive loops corresponding to the three timepoints in the stepped wedge design at which groups of NICUs entered the implementation phase of the Neo-MILK intervention. In each loop, SSM was applied to the five NICUs each that had most recently transitioned from the control to the intervention phase (loop 1: April 2022; loop 2: October 2022; loop 3: April 2023). In this paper, we focus on the first loop as an illustrativeexample to demonstrate the SSM-MMCS methodology in detail.

#### Setting

The c-RCT was carried out in 15 NICUs across Germany. Clinics from different federal states, of different sizes, with different ownership structures and with departments relevant to the study (i.e. level I NICUs) were included. Each participating clinic had to give its consent by signing a cooperation agreement.

**Fig. 1:**
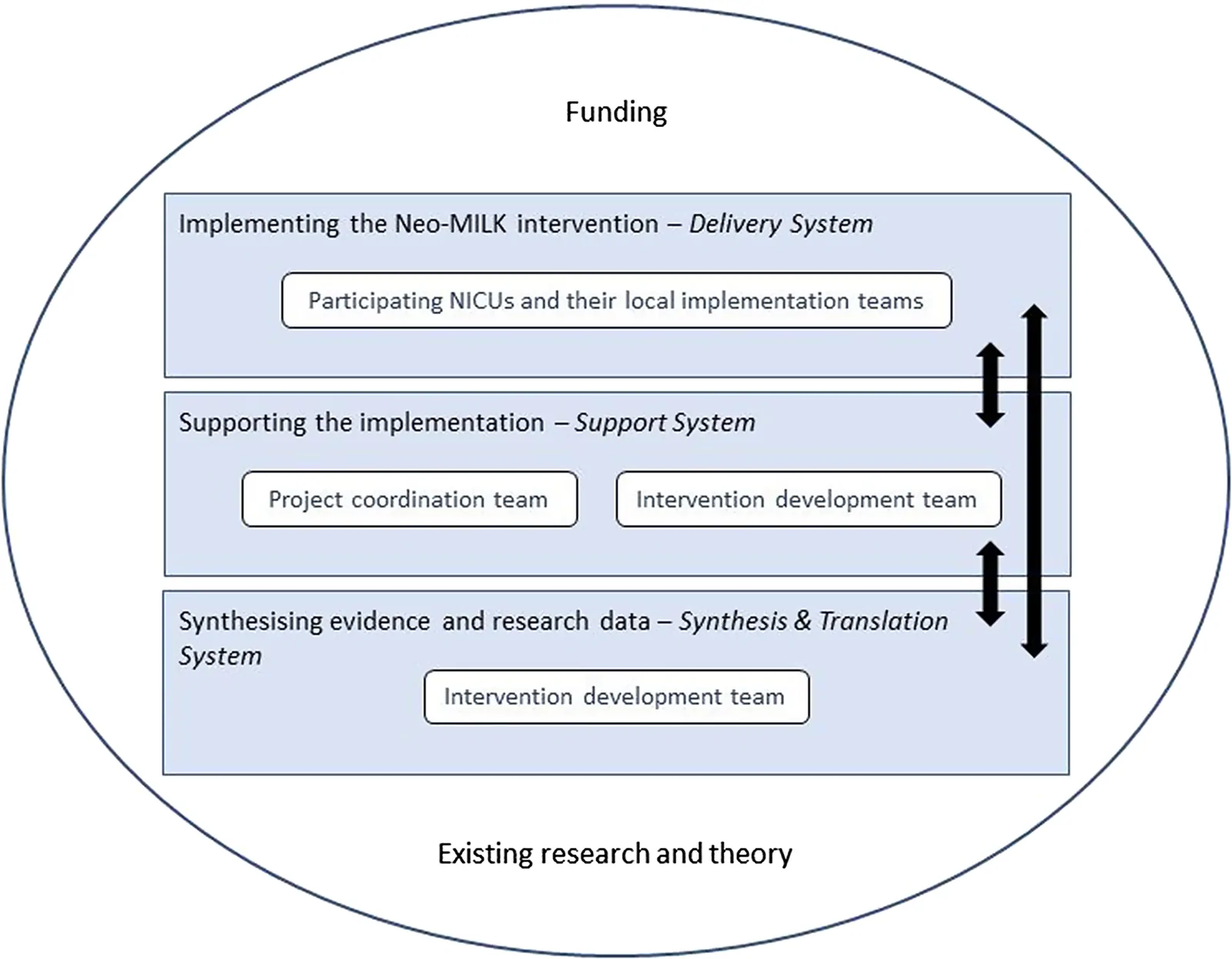
Neo-MILK implementation system based on the interactive systems framework for dissemination and implementation [45]

To described the implementation system within which the Neo-MILK intervention was deployed and to provide a greater understanding of how the EBI was implemented, we drew on the Interactive Systems Framework for Dissemination and Implementation (ISF) by Wandersman et al. [35] (Fig. 1). The ISF distinguishes three core systems: the *synthesis & translation system*, which distils research evidence into implementable interventions and strategies; the *support system*, which provides training, technical assistance, and resources to facilitate implementation; and the *delivery system*, which carries out the implementation of the intervention in practice. Applying this framework to the Neo-MILK project, the implementation system can be described as follows: Each NICU, representing a *delivery system*, was independently responsible for implementing the EBI in their routine practices. Local implementation teams organised the implementation process to align with their specific context and workflow. The implementation was supported by a *support system*. Following the site initiation visit, the project coordination team provided training and informational materials tailored for the NICU staff as well as guidelines, procedural policies, and nudging (initial implementation strategies). Together with the intervention development team the project coordination team addressed any emerging inquiries, ensuring that the NICUs had access to necessary information and guidance to successfully implement the intervention. The intervention components, materials, and initial implementation strategies were developed by the intervention development team before the study. Within the IFS the intervention development team is referred to as the *synthesis & translation system*. By synthesising evidence and research data, the intervention development team ensured that the intervention was both evidence-based and contextually relevant. In addition, the initial implementation strategies were designed to address needs that were identified through a national survey [32]. Furthermore, the project was funded by the Innovation Fund of the Federal Joint Committee (*Gemeinsamer Bundesausschuss*) (grant number 01NVF19027).

### Application of the system support mapping and matrixed multiple case study approach

We used SSM as a core methodology as part of the progress-focused FE [36] of the Neo-MILK project to explore the context of the participating NICUs and to identify their local implementation determinants.

Previous literature has mainly described the use of SSM as a self-reporting tool for individuals or implementation teams (see Additional file 1). In contrast, we used it as a method to analyse the implementation progress perceived by healthcare professionals at the NICUs and collected data through semi-structured interviews during each of the three loops of the FE. Specifically, we applied SSM to identify context-specific factors that facilitate or inhibit implementation. We embedded SSM in a process comprising six steps:

1. *Gather data on the implementation process*.
2. *Assess implementation barriers and facilitators per key person and NICU*.
3. *Group data into categories at key person level over all NICUs*.
4. *Organise the categories into a sortable matrix*.
5. *Conduct within-site analysis of matrixed data*.
6. *Conduct cross-site analysis of matrixed data*.

**Fig. 2:**
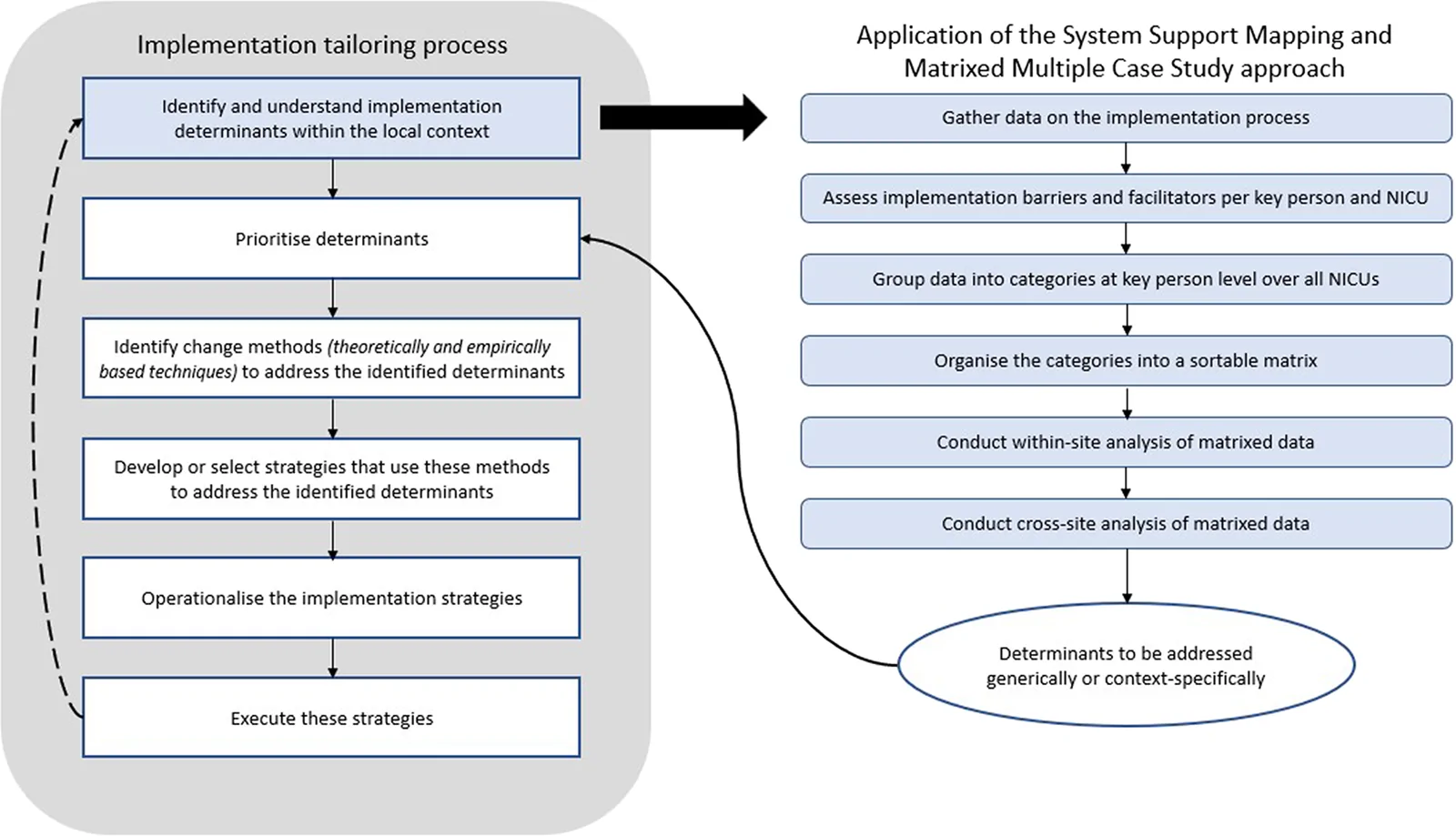
Conceptual positioning of the system support mapping and matrixed multiple case study approach within the implementation tailoring process

Steps 1 to 3 are primarily informed by the SSM approach and involve data collection using SSM-informed interview guides (Step 1), the coding of data according to the SSM elements and mapping of interrelationships (Step 2), and the grouping of codes into analytically comparable categories (Step 3). Steps 4 to 6 integrate the MMCS approach, whereby the SSM-generated categories are organised into a sortable matrix (Step 4), and within-site (Step 5) and cross-site (Step 6) comparisons are conducted using the matrix structure proposed by Kim et al. [26]. The outputs of this process, which consist of structured matrices showing the perceived impact of implementation determinants within and across sites, may serve as a basis for subsequent steps in the implementation tailoring process, such as the selection or adaptation of implementation strategies (see Fig. 2 for the conceptual positioning of the methodology within the broader tailoring process). While this study focuses on demonstrating steps 1 to 6 as a methodology for structured context assessment, the generated outputs may inform subsequent tailoring steps, which remain to be investigated in future research.

#### Step 1: Gather data on the implementation process

We conducted semi-structured interviews by video call or telephone with up to two key persons from each NICU (including a pre-test interview) during their working time. As we gathered data qualitatively, we used the Consolidated Criteria for Reporting Qualitative Research (COREQ) checklist [37] (Additional file 2). The interview guideline was developed by ND and JKN. It was designed to elicit the information required for the SSM-based analysis presented here and, at the same time, to serve the process evaluation of the c-RCT (the interview guideline translated into English, annotated with the SSM element(s) addressed by each question where applicable, can be found in the Additional file 3). Because of this dual purpose, not every question was constructed to address a particular SSM element. Questions were phrased in the everyday professional language of the interviewees, with the SSM elements applied as analytical categories during the subsequent analysis (step 2). The annotations in Additional file 3 therefore indicate which element(s) a question was primarily intended to inform rather than a one-to-one correspondence between question wording and element label. Researchers adapting this guideline should therefore ensure that the questions collectively cover all five SSM elements. Key persons were healthcare professionals (nurses and physicians) with in-depth knowledge of NICU workflows and organisational structures who held local project responsibility and/or were actively and operationally involved in the implementation of the Neo-MILK intervention at their respective NICU. Wherever possible, we conducted interviews with two key persons per NICU to capture both strategic and operational perspectives. Modifications of the interview guideline after a pre-test interview were minor, only concerning the sequence and the rephrasing of a few questions. After two months of the intervention phase, one female researcher (ND), who had basic experience in qualitative health research and an educational background in health economics and healthcare management, conducted interviews to identify emerging barriers and facilitators during the ongoing implementation process. The interviews lasted between 45 and 60 min. ND received feedback about her interviewing techniques from JKN after the pilot interview.

At the beginning of each interview, a shared understanding of implementation success was established. Subsequently, the following topics were explored in greater depth: expectations regarding the project and its project partners, the status quo of care before the intervention phase, implementation preparation, and perceived status quo of the implementation process. Informed consent was obtained from all participating key persons.

#### Step 2: Assess implementation barriers and facilitators per key person and NICU

After transcribing the audio-recorded interviews and checking for accuracy [38], each interview was analysed using directed content analysis [39]. The SSM elements, consisting of an individual’s perceived *role(s)* and their *responsibilities*, *needs*, *resources*, and *wishes*, served as pre-defined deductive categories (see Additional file 1 and Table 1). Within these deductive categories (hereinafter referred to as SSM elements), inductive coding was applied to develop context-specific codes reflecting the particular circumstances and perspectives reported by each key person. This combined approach was chosen because the SSM elements provide a theoretically grounded structure for organising the data, while inductive coding within each element allows for the identification of locally relevant factors that cannot be anticipated a priori. This approach is consistent with directed content analysis as described by Hsieh and Shannon [39], in which existing theory provides the initial coding scheme and additional codes emerge from the data.

Codes for each SSM element were initially developed by one member of the research team (ND) and subsequently reviewed by a second member (JJ). Both coders (health economists) then jointly revised the codes and reconciled disagreements. The results were presented and discussed during interprofessional meetings (with AS, DW, TD, and NS) to secure intersubjective comprehensibility and credibility. We decided to use a pragmatic rapid analysis approach [40, 41] to allow for rapid sense-making and problem-solving by timely sharing critical information within the implementation process. To capture the full breadth of local factors, it was deemed preferable to refrain from utilising a determinant framework for coding during this step. Data management and coding were supported by MAXQDA (version 20.4.2) [42].

**Fig. 3:**
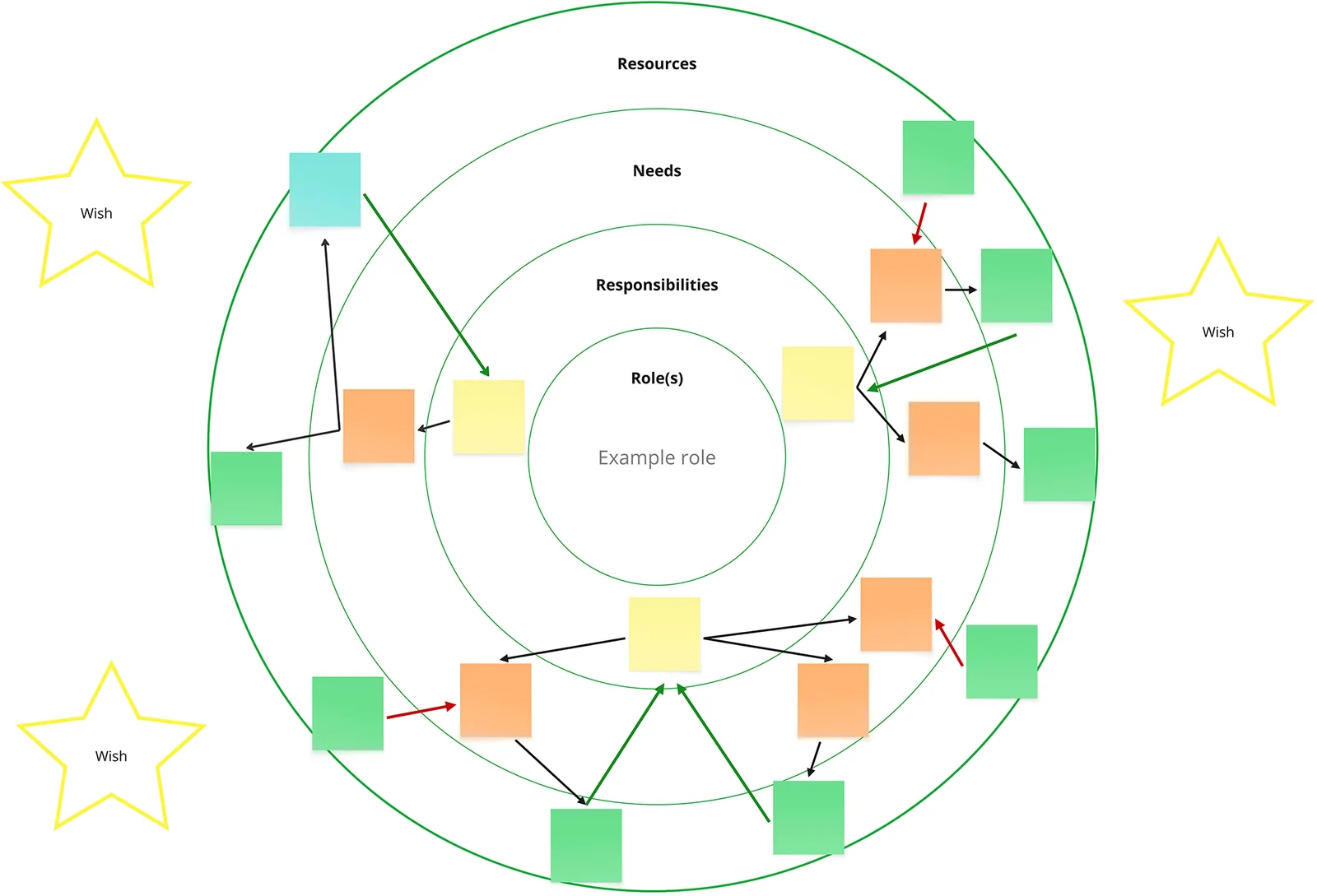
Example system support map (SSM) without content to illustrate its visualisation. Note. SSM elements are displayed as follows: role(s) in written form, responsibilities in yellow rectangles, needs in orange rectangles, organisational resources in green rectangles, personal resources in blue rectangles, and wishes in gold stars. The arrows indicate that the arrow source is connected to the element the arrow points to. Green arrows indicate a facilitating impact, and red arrows indicate an inhibiting impact. Connections with no reported impact are shown as black arrows

Furthermore, the interrelationships between the inductively developed codes across SSM elements were examined, with the aim of exploring which factors were perceived by a key person to influence the implementation of the intervention. Interrelationships were coded when a key person explicitly described a connection between elements and its perceived impact on the implementation process. Valence assignment followed a consistent scheme in which a connection was coded as facilitating if a code within one SSM element was described as enabling or supporting the fulfilment of codes in another element (e.g., a resource enabling the fulfilment of a responsibility); as inhibiting if the code was described as preventing, constraining, or falling short of what was needed; and as having no directional impact on implementation if a connection was identified but no facilitating or inhibiting effect was reported. These interrelationships were depicted using directional links (arrows) between codes, as illustrated in Fig. 3. Connections with no impact are shown as black arrows. Connections with a facilitating effect on implementation are highlighted in green. Inhibiting interdependencies, however, are labelled red. As the interviews were focused on wishes regarding the implementation process and the project in general, we refrained from linking the *wishes* category with others. The coding scheme for connections between SSM elements is describes in Table 1. Interrelationship coding was also carried out by one researcher (ND) and subsequently reviewed by a second researcher (JJ); disagreements were reconciled through discussion, following the same reconciliation process applied to the code development.

**Table 1:** Core elements of system support mapping (SSM) and their definitions as applied in this study

SSM element	Definition	Application in this study
Role(s)	The function(s) an individual perceives to hold within the implementation process	Used as a contextual frame to understand the perspective from which responsibilities, needs, resources, and wishes are reported
Responsibilities	Tasks and duties associated with an individual’s role in the implementation process	Coded inductively within the deductive SSM category to identify specific implementation-related tasks
Needs	Requirements that must be met for an individual to fulfil their responsibilities	Coded inductively to identify unmet needs that may constitute implementation barriers
Resources	Material (tangible) and non-material (intangible) assets available to an individual	Coded inductively; identified as the SSM element most critically linked to facilitating and inhibiting factors
Wishes	Desired changes or improvements expressed by an individual regarding the implementation process	Coded inductively; not linked to other elements, as interviews focused on wishes regarding the overall project
Interrelationship between elements	Directional connections between codes across SSM elements, reflecting a perceived impact of one element on another.	Coded as facilitating (depicted as green arrows) if a code in one element was described as enabling or supporting the fulfilment of codes in another; as inhibiting (depicted as red arrows) if described as preventing, constraining, or falling short of what was needed; and as having no directional impact (depicted as black arrows) if a connection was identified but no facilitating or inhibiting effect was reported.

Note. SSM = System Support Mapping; Definitions are based on Calancie et al. [23] and have been adapted for the context of this study. See Additional file 1 for a detailed description of SSM

#### Step 3: Group data into categories at key person level over all NICUs

To be able to compare the individual content within a NICU and across all NICUs in the subsequent steps, the inductively developed codes of the respective SSM elements were sorted and grouped into categories by using axial coding [43]. The categories were developed at key person level (i.e. separately for key persons with strategic project responsibility and key persons with operational involvement in the implementation of the intervention) to allow consideration of potential differences between the types of key persons. The axial coding was carried out independently by two researchers, after which the categories were revised by the coding team (ND and JJ) and discrepancies reconciled. The categories are listed in the Supplementary Tables 3 and comprise examples of the inductively developed codes within the respective SSM elements from step 2, from which the categories were derived. As the interviews were conducted in German, the inductively formulated codes and the resulting categories were translated into English. The translation followed the TRAPD (Translation, Review, Adjudication, Pretest, Documentation) approach [44].

While all five SSM elements were coded and grouped into categories, the subsequent analysis (steps 4–6) may focus on specific SSM elements, representing a methodological choice within the six step-approach. This choice can be guided by different criteria, such as the empirical prominence of an element in the interrelationship analysis (as in the present case), the research question, or theoretical considerations.

#### Step 4: Organise the categories into a sortable matrix

**Fig. 4:**
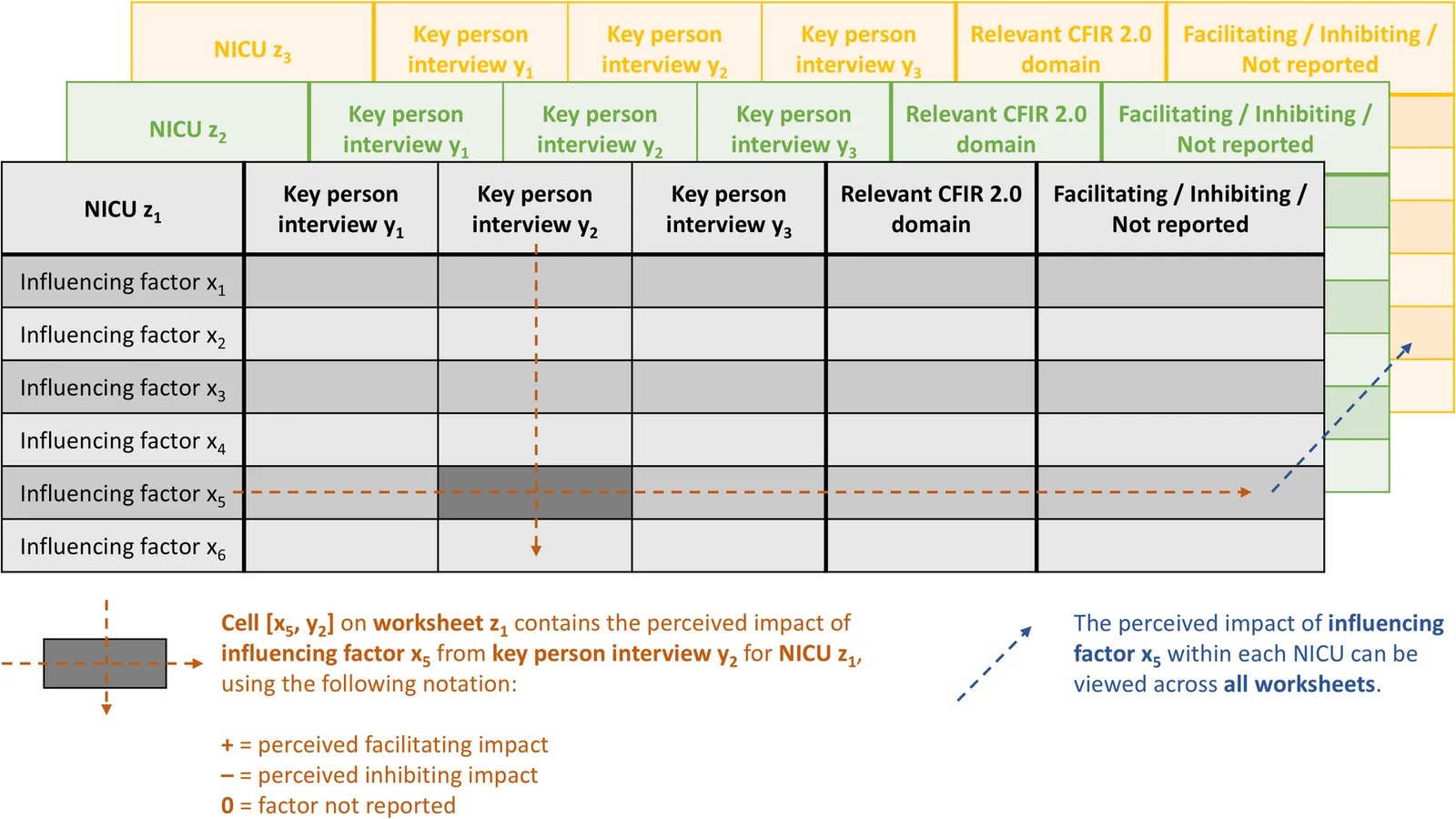
Matrixed organisation of multi-site data based on Kim et al. [27]

The categories identified as influencing factors (based on the identified connections between the inductive codes of the various SSM elements and their perceived impacts in step 2) were organised into a matrix, in which each column indicated a data source (an interview with a key person), each row indicated an influencing factor, and each raw-by-column table (worksheet) represented a NICU, as suggested in the MMCS approach. Figure 4 illustrates this matrix structure and its analytical logic (for further detail see the work from Kim et al. [26]. The organisation of data in this manner confers a distinct advantage, as it permits the matrix to be sorted according to three distinct criteria: NICU, key person interview and influencing factor. This enables the data to be grouped together, facilitating comprehensive within-site and cross-site comparisons and analyses (steps 5 and 6). For each influencing factor included in the matrix, we additionally designated a domain (outer setting domain, inner setting domain, individuals domain, implementation process domain) using the updated CFIR 2.0 [45] to allow for a better grouping of factors. We noted the perceived impact of each influencing factor for each key person and used “+” as an indication for a facilitating impact, “−” to denote an inhibiting impact, and “0” if the determinant was not reported or if there was no impact reported on the implementation of the intervention.

#### Step 5: Conduct within-site analysis of matrixed data

We examined the data on each influencing factor across all key persons per NICU. Subsequently, each factor was designated as either reported or not reported and having either a perceived facilitating and/or inhibiting impact on the implementation process using the same labels as previously mentioned (step 4). One member of the research team (ND) summarised the data for each NICU. Following this, a second member of the research team (JJ) was consulted to provide their input on the labelling to finalise the designations.

#### Step 6: Conduct cross-site analysis of matrixed data

Using the sortable matrix, we first assessed whether the listed influencing factors were reported across all NICUs (i.e., worksheets). Secondly, we assessed their reported perceived impact on the implementation process across all NICUs. This also involved noting which factor had common versus distinct perceived impacts at different sites (e.g., a factor had a perceived facilitating impact at all NICUs, while another factor had a perceived facilitating impact at some NICUs and a perceived inhibiting impact at other NICUs). Then, cross-site trends were identified and characterised as follows: *homogeneously facilitating*,* homogeneously inhibiting*,* heterogeneous – predominantly facilitating*, or *heterogeneous – predominantly inhibiting*. When reported, factors were characterised as having a homogeneous impact if they were perceived uniformly across all NICUs as having a facilitating or inhibiting effect. If the impact of a factor was perceived to be heterogeneous across NICUs, the factor was characterised as having a predominantly facilitating or inhibiting impact, depending on whether the number of positive perceptions or the number of inhibiting perceptions predominated. This characterisation is of particular interest, as it provides insight into the homogeneity of influencing factors across different sites. It may indicate whether the use of cross-site implementation strategies is appropriate or whether strategies need to be adapted to the local context and challenges.

## Results

In the following, we present the six-step SSM-MMCS methodology using data from the first loop of the Neo-MILK FE as an illustrative example. Results are structured according to each methodological step, illustrating both the procedural logic and the type of output it generates, with selected findings as examples to make visible the insights the approach can surface.

### Step 1: Gather data on the implementation process

A set of audio-recorded and transcribed interviews provided the raw data for the subsequent SSM-based analysis. The number of interviews per site, the key person characteristics (in our case strategic versus operational involvement), and the thematic focus of the interview guide may determine the richness and scope of the evidence base that steps 2 and 3 can draw upon. In this application, we conducted a total of 10 interviews with key persons from the five NICUs of the first randomised group moving from the control phase to the intervention phase. Key persons were nurses and physicians with in-depth knowledge of workflows and organisational structures and were actively involved in the implementation of the EBI. On average, they had been in their current job position for 7 years and reported 15 years of working in a NICU setting.

### Step 2: Assess implementation barriers and facilitators per key person and NICU

**Fig. 5:**
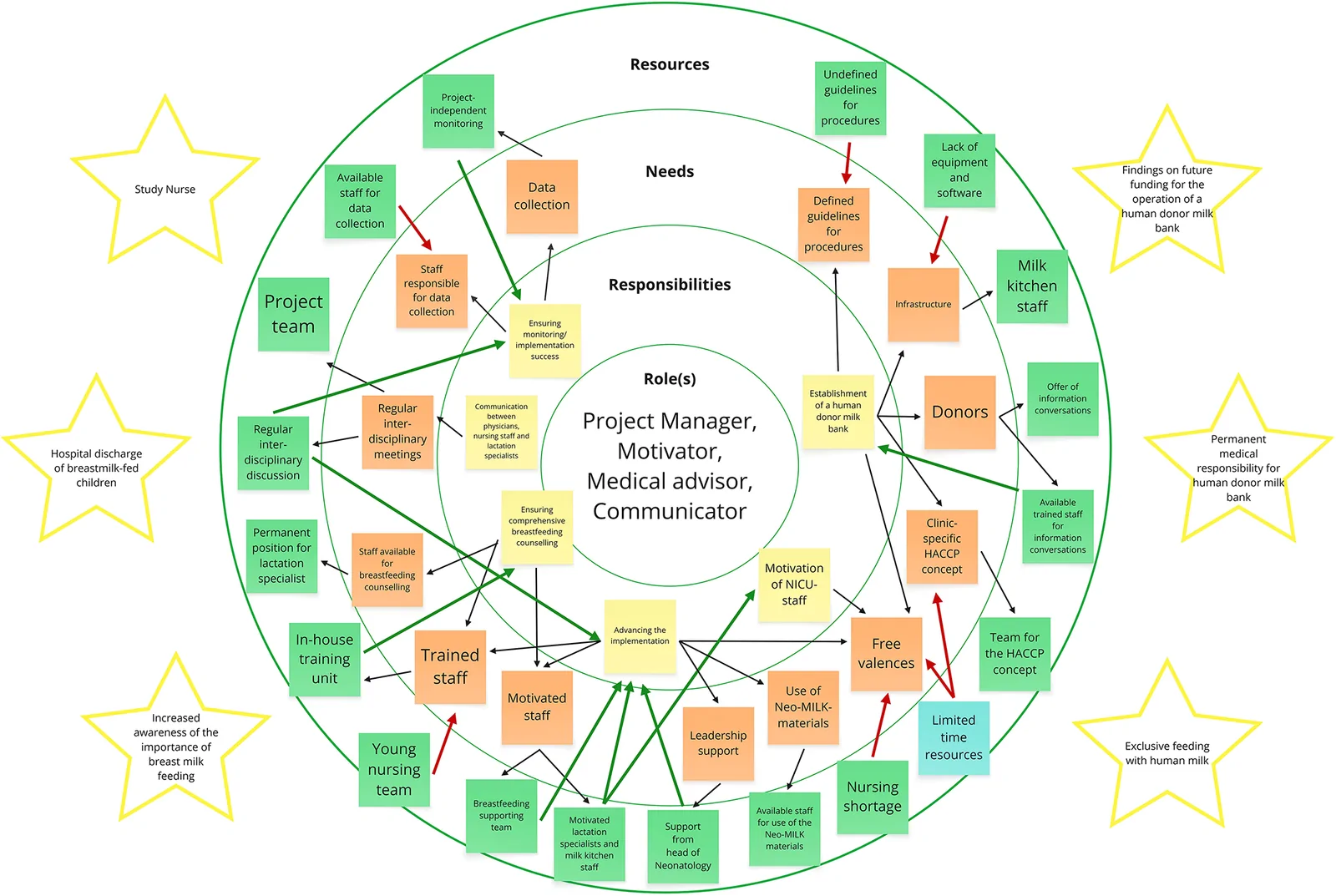
Example system support map (SSM) based on one key person interview. Note. Inductively developed codes within the SSM elements are displayed as follows: codes for role(s) in written form, codes for responsibilities in yellow rectangles, codes for needs in orange rectangles, codes for organisational resources in green rectangles, codes for personal resources in blue rectangles, and codes for wishes in gold stars. The arrows indicate that the arrow source is connected to the element the arrow points to. Green arrows indicate a facilitating impact, and red arrows indicate an inhibiting impact. Connections with no reported impact are shown as black arrows

Using the inductively developed codes within the deductive categories given by the SSM elements, we created individual maps for each key person of each NICU (see Fig. 5 for an illustrative example). These maps visualise the interrelationships between an individual’s role(s), responsibilities, needs, resources, and wishes, with green arrows indicating a perceived facilitating impact and red arrows indicating a perceived inhibiting impact on the implementation process. Across all maps, the SSM element *resources* emerged as the most prominent factor influencing implementation. Applying the selection criterion outlined in the Methods section, the subsequent analysis therefore focused on this element. This reflects an analytically motivated adaptation of the approach; other applications may identify different SSM elements as the primary focus, depending on the patterns emerging from the individual maps. *Resources* were connected to both responsibilities and needs. The perceived impact of reported resources depended on whether they enabled the fulfilment of reported responsibilities (facilitating) or fell short of addressing the reported needs required for successful implementation (inhibiting). Both tangible resources (e.g., staff, equipment, infrastructure) and intangible resources (e.g., motivation of NICU staff, communication, leadership support) on organisational and personal level were identified as influencing factors. For example, one key person with a primarily operational role highlighted infrastructural uncertainty as a tangible resource constraint: “*I think we’ll be moving into our new building next year*,* where hopefully our milk kitchen will also be located*,* though that’s not entirely certain*,* as you don’t think of everything when planning a new building.*” (I1). In contrast, a key person with a strategic role emphasised the importance of structured communication as an intangible resource within the same NICU: “*I do think we should schedule regular meetings*,* once a month. […] And I think that makes sense too*,* because then we can keep discussing the current situation.*” (I2).

### Step 3: Group data into categories at key person level over all NICUs

Inductively developed codes were grouped into analytically comparable categories within each SSM element covering all five elements (roles, responsibilities, needs, resources, and wishes) to enable systematic comparison within and across sites in the subsequent steps. Initially, the grouping was intended to be conducted at key person level. However, due to the similarities in reported *responsibilities*, *needs*, and *resources*, this separation was deemed unnecessary. Key persons with a rather strategic involvement in the implementation process (self-reported) identified their *roles* as local project lead, coordinator, motivator, and supervisory authority. Key persons with a more operational involvement (self-reported) also reported their roles to be that of a coordinator, motivator, supervisory authority, and an operational implementation instance.

Across all sites we developed 21 initial codes within the element of *responsibilities* and grouped them into five categories, such as enhancing knowledge and skills of NICU staff and management and monitoring of the implementation process. From 38 inductively coded *needs*, we developed 11 *needs* categories, including leadership support, motivation and positive attitude of NICU staff towards the implementation of the Neo-MILK project content, and staff and equipment resources for the deployment of lactation and breastfeeding support. The 93 codes developed within the *resources* SSM element were grouped into 16 categories, including among others, staff resources for organisational and content-related project activities and infrastructural conditions (tangible resources), as well as regular dialogue within the implementation team involving the disciplines concerned and motivation and attitudes of NICU staff towards the implementation of the intervention components (intangible resources). Furthermore, we identified 32 codes as *wishes* related to the implementation process and the overall project across all sites and developed eight categories such as support from policymakers, increase in staff with permanent responsibilities, and standardised knowledge of pumping, breastfeeding and HDM among NICU staff to provide comprehensive advice to mothers/parents.

Table 2 lists all the categories developed within each SSM element. In addition, Supplementary Table 3 contains a list of all categories developed by axial coding within the different SSM elements, together with an exemplary selection of up to three codes from which the categories were derived. As the patterns identified in step 2 indicated *resources* as the analytically most salient element influencing implementation, the categories developed within this element served as the primary basis for the MMCS-based analysis in steps 4 to 6.

**Table 2:** Categories developed from initial codes within each SSM element

SSM element	Categories within each SSM element
Role(s)	Local project lead Coordinator Motivator Supervisory authority Operational implementation instance
Responsibilities	Establishment of a HDMB Enhancing knowledge and skills of NICU staff Motivation of NICU staff Management and monitoring of the implementation process Implementation of the elements of the lactation and breastfeeding support programme
Needs	Leadership support Motivation and positive attitude of NICU staff towards the implementation of the Neo-MILK project content Standardised know-how among NICU staff Reliable communication structures within the implementation team involving the disciplines concerned Database for monitoring Staff and equipment resources for the deployment of lactation and breastfeeding support Time resources available in the teams Management of knowledge within the service-providing team and between different team structures Clinic-specific defined SOPs/instructions for operations and procedures Infrastructure for operating a HDMB Collection of HDM
Resources	NICU internal training offers Regular dialogue within the implementation team involving the disciplines concerned Leadership support Staff resources for organisational and content-related project activities Infrastructural conditions External drivers Scarce time resources for project activities due to high workload in everyday NICU life Personal resources Motivation and attitudes of NICU staff towards the implementation of the intervention components Extensive process of HDM collection with restrictions Development stage of clinic-specific SOPs/instructions for operations and procedures Resources generated by initial implementation strategies Knowledge of Neo-MILK project content Permanent positions for lactation and breastfeeding counselling in the NICU Established documentation routines and monitoring processes Internal clinic guidelines
Wishes	Support from policymakers Regulatory authorities and professional associations Enhanced collaboration through improved awareness of the importance of breast milk feeding Exclusive feeding with breast milk, alternatively with HDM Increase in staff with permanent responsibilities Standardised knowledge of pumping, breastfeeding and HDM among NICU staff to provide comprehensive advice to mothers/parents Sustainable integration of lactation and breastfeeding-promoting Neo-MILK content into everyday working life Database for monitoring, process optimisation and quality assurance

Note. HDM = human donor milk, HDMB = human donor milk bank, NICU = neonatal intensive care unit, SOP = standard operating procedure

### Step 4: Organise the categories into a sortable matrix

We organised the developed *resource* categories mentioned in step 3 into a sortable matrix and noted the perceived impact of each influencing factor per key person. Depending on the individual responsibilities, needs, and context, *resources* were perceived as inhibiting or facilitating factors, but in some cases, they were not reported at all if the respective factor was not relevant.

As we aimed to allow a better grouping of these factors, we used CFIR 2.0 [45] and assigned a domain for each influencing factor, resulting in the following allocation:

- **Inner setting domain**: Development stage of clinic-specific standard operating procedures (SOPs) or instructions for operations and procedures; established documentation routines and monitoring processes; extensive process of HDM collection with restriction; infrastructural conditions; internal clinical guidelines; leadership support; NICU internal training offers; regular dialogue within the implementation team involving the disciplines concerned; scarce time resources for project activities due to high workload in everyday NICU life; staff resources for organizational and content-related project activities.
- **Outer setting domain**: External drivers.
- **Individuals domain**: Knowledge of Neo-MILK project content; motivation and attitudes of NICU staff towards the implementation of the intervention components; personal resources.
- **Implementation process domain**: Permanent positions for lactation and breastfeeding counselling in the NICU; resources generated by initial implementation strategies.

This grouping illustrates that most of the implementation determinants are situated within the inner setting, with only a few being external, individual or implementation process-related.

### Step 5: Conduct within-site analysis of matrixed data

Table 3 summarises the within-site analysis outputs for all five NICUs with the reported determinants being perceived as facilitating and/or inhibiting within each NICU. It should be noted that the initially planned number of key persons per NICU was two. Three interviews were conducted in NICU 3, as two persons shared the same tasks and responsibilities, and only one interview in NICU 4, as no second key person agreed to be interviewed. The within-site analysis revealed some noteworthy patterns. First, the influencing factor, i.e. the content of the element *resources*, can have both a facilitating and inhibiting influence within the same NICU, depending on either the perception of the key person or the many nuances of these *resources* categories (see Supplementary Table 3 for the codes for each category). For example, in NICU 4, *Staff resources for organisational and content-related project activities* was perceived as both facilitating and inhibiting by the same key person, because this resource supported some responsibilities while being insufficient for others. This dual perception is illustrated by the following statement from one interviewee, who noted the impact of task delegation while simultaneously perceiving overall staffing levels as an inhibiting constraint: “*As a large clinic*,* we have some shifts that are now staffed by just three people. […] This will likely undermine the impact we could achieve with all the excellent intervention components on offer.*” (I8). Second, the analysis showed that not all determinants were reported by all NICUs, which may reflect differences in local contexts, implementation progress, and the individual needs of the interviewed key persons rather than the absence of these determinants per se. Third, the linking of resources to responsibilities and needs through the SSM approach may provide an explanation for why a resource was perceived as facilitating or inhibiting. For instance, leadership support was reported as facilitating when it enabled the allocation of dedicated time for implementation activities but was perceived as less relevant in NICUs where implementation was primarily driven by individual commitment rather than formal leadership directives. For example, one key person emphasised the dependency on formal support structures: “*I think it would be difficult for the team to put this into practice if the leadership isn’t fully supporting it.*” (I5).

**Table 3:** Perceived impact within and across NICUs for reported resources categories

Facilitating and/or inhibiting *resources* categories reported across all NICUs	Perceived impact within each NICU ( + = facilitating, − = inhibiting, 0 = not reported)					Perceived impact across all NICUs
	NICU 1	NICU 2	NICU 3	NICU 4	NICU 5	
Inner setting domain						
Development stage of clinic-specific standard operating procedures/instructions for operations and procedures	0	**−**	0	0	**−**	homogeneously inhibiting
Established documentation routines and monitoring processes	**+**	**+**	**+**	**+**	**+**	homogeneously facilitating
Extensive process of HDM collection with restrictions	**−**	**+**	**−**	**−**	0	heterogeneous – predominantly inhibiting
Infrastructural conditions	**+** / **−**	**−**	**+** / **−**	0	**−**	heterogeneous – predominantly inhibiting
Internal clinical guidelines	0	**−**	**−**	0	**−**	homogeneously inhibiting
Leadership support	0	**+** / **−**	**+**	0	**+**	heterogeneous – predominantly facilitating
NICU internal training offers	**+**	**+**	**+**	0	**+**	homogeneously facilitating
Regular dialogue within the implementation team involving the disciplines concerned	**+**	**+**	**+**	**+**	**+**	homogeneously facilitating
Scarce time resources for project activities due to high workload in everyday NICU life	**−**	**−**	**−**	**−**	**−**	homogeneously inhibiting
Staff resources for organisational and content-related project activities	**+** / **−**	0	**+** / **−**	**+** / **−**	**−**	heterogeneous – predominantly inhibiting
Outer setting domain						
External drivers	**+**	**−**	**+** / **−**	0	**−**	heterogeneous – predominantly inhibiting
Individuals domain						
Knowledge of Neo-MILK project content	**−**	**+** / **−**	**+** / **−**	0	0	heterogeneous – predominantly inhibiting
Motivation and attitudes of NICU staff towards the implementation of the intervention components	**−**	**+** / **−**	**+** / **−**	**−**	**+**	heterogeneous – predominantly inhibiting
Personal resources	**+**	0	**+**	**+**	**−**	heterogeneous - predominantly facilitating
Implementation process domain						
Permanent positions for lactation and breastfeeding counselling in the NICU	**+**	**+** / **−**	0	**−**	**+**	heterogeneous – predominantly facilitating
Resources generated by initial implementation strategies	**+**	**+**	**+**	**+**	**+**	homogeneously facilitating

Note. HDM = human donor milk, NICU = neonatal intensive care unit

Additional file 4 illustrates the different impacts of all reported resources by showing the mode of action of each *resource* by linking them to *responsibilities* and *needs*. The thicker the arrow, the more frequently this interrelationship was reported in the interviews conducted across all sites.

### Step 6: Conduct cross-site analysis of matrixed data

To conduct the cross-site analysis, we applied the sorting capability of the matrix from step 4: for each influencing factor (row), we read across all NICUs (worksheets) and synthesised the reported perceived impacts, classifying each determinant as showing either a homogeneous (consistent perceived impact across reporting sites) or heterogeneous pattern (varying perceived impact across sites). This distinction is summarised in the *Perceived impact across all NICUs* column of Table 3.

With regard to homogeneous patterns, it is noteworthy that four out of the five *resources* categories that were collectively considered to have a homogeneous impact were reported by all sites. It was therefore perceived by all NICUs that the *resources* categories *established documentation routines and monitoring processes*, *regular dialogue within the implementation team involving the disciplines concerned*, and *resources generated by initial implementation strategies* had a facilitating impact. In contrast, *scarce time resources for project activities due to high workload in everyday NICU life* were perceived as an inhibiting factor across all five NICUs. A pattern that reflects broader structural challenges in clinical settings, as one key person describes: “*There’s a staff shortage at the moment. We’ve been in what I’d call an absolute emergency situation in the hospitals for the last two or three weeks and we don’t know […] how we’re ever going to manage the workload.*” (I9). These homogeneous patterns may suggest that certain facilitators and barriers operate relatively independently of local context and may therefore be amenable to cross-site implementation strategies. In contrast, the overall impact of other *resources* categories was observed to be heterogeneously perceived, manifesting as either predominantly facilitating or predominantly inhibiting factors. For example, *resources* category *infrastructural condition* was reported as predominantly inhibiting, but were perceived as facilitating in NICUs where spatial arrangements supported the implementation of a human donor milk bank. Similarly, *motivation and attitudes of NICU staff towards the implementation* showed variation across sites, with some NICUs reporting high intrinsic motivation while others described resistance or ambivalence among parts of the staff. For example, a key person at one NICU observed a lack of prioritisation among specialist staff: “*They always go on about how ventilation is important*,* or how resuscitation is important*,* but somehow they don’t seem to think breast milk is important.*” (I3). These heterogeneous patterns indicate that the same nominally identical determinant (e.g., “infrastructural conditions”) may manifest differently across sites due to differences in local context, highlighting the need for site-specific assessment and tailored responses.

## Discussion

In this paper, we described and illustrated the combined application of SSM and the MMCS approach as a structured six-step methodology for identifying and analysing implementation determinants within and across sites. Using data from the first loop of the progress-focused FE in the Neo-MILK project, we demonstrated how SSM can be used as an interview-based analytical methodology, extending its original use as a self-reporting tool. We also showed how its outputs can be systematically organised, compared, and synthesised using the MMCS approach.

### Methodological contributions and reflections

Several methodological insights emerged from this application. SSM can be regarded as a useful analytical framework for organising qualitative data around the role(s), responsibilities, needs, resources, and wishes of individual interest-holders within one system. By mapping the interrelationships between these elements, the approach made visible which factors were perceived to influence implementation. By revealing that *resources* can be perceived as facilitating for specific *responsibilities* and simultaneously be perceived as insufficient for related *needs*, SSM has the ability to provide a holistic understanding of a system and its dynamics. While the SSM structure captures which factors are perceived to influence implementation and in what direction, the underlying interview material on which a map is based provides an additional layer of contextualisation. By drawing on this material, this approach may shed light not only on what influences implementation, but also on how and why specific determinants are perceived as facilitating or inhibiting within a given context. This mechanism-oriented perspective may additionally support the anticipation of potential ripple effects, that is, how a change in one element of the system may affect other interrelated elements. Such insights align with calls in the implementation science literature for moving beyond cataloguing determinants towards understanding their mechanisms of action [46, 47]. It should be noted that the decision to focus the cross-site analysis on the SSM element resources was an empirically motivated methodological choice rather than an inherent constraint of the approach. The six-step methodology allows for the inclusion of all SSM elements in steps 4–6, and the decision regarding which elements to carry through into the matrix-based comparison can be adapted depending on the research question, the complexity of the data, and the specific aims of the analysis.

However, while SSM generates rich, case-specific data, it does not inherently provide a structure for systematic comparison across cases. The MMCS approach complemented SSM in this regard. The MMCS approach enabled us to organise the SSM-generated categories into a sortable matrix and therewith to identify both homogeneous and heterogeneous patterns in the perceived impact of determinants across NICUs, a distinction with implications for the design of cross-site versus site-specific implementation strategies.

### Cross-site determinant patterns

A synthesis of the findings indicates that, in addition to tangible *resources* (i.e. material, such as equipment) also intangible *resources* (i.e. nonmaterial, such as psychological safety) are critical to the implementation of EBIs. This is consistent with findings from other implementation studies highlighting the role of organisational resources and capacities in shaping implementation success [45, 48]. The within-site analysis revealed that the perceived impact of a given *resources* category depended on the specific *responsibilities* and *needs* of individual interest-holders, underscoring the value of capturing multiple perspectives within each site. By making these interrelationships visible, SSM may enable implementation teams to examine not only which determinants are present, but also the genesis of these determinants, providing an important starting point for context-sensitive assessment.

The cross-site analysis further demonstrated that nominally identical determinants may manifest differently across sites. Determinants with homogeneous impacts across NICUs may be amenable to standardised cross-site strategies, enabling the efficient use of implementation resources, the bundling of capacities, and the reinforcement of collective efforts during an implementation process. This is due to the fact that implementation strategies addressing determinants with a homogeneous genesis and impact across implementation sites can be designed at a more generic level. In this context, the call for more detailed reporting of determinants in the literature appears to be warranted. The reporting of determinants, and in particular their potentially different genesis in the implementing sites, could provide valuable information for new implementation initiatives, enabling the distribution of implementation resources between the higher and local implementation levels to be optimised.

Determinants with heterogenous relevance, impact or genesis across sites, remain the responsibility of local implementation teams. The heterogeneous perceptions of the impact of a *resources* category within a site and across sites may be attributed not only to the multitude of nuances pertaining to the responsibilities and needs of various individuals and their roles, but also to the differences in the nuances of the *resources* themselves. A determinant may carry the same name at a superordinate level, yet differ substantively in one or more sites in interaction with the respective context, highlighting the importance of tailoring implementation [49]. The extent of homogeneous and heterogeneous genesis of determinants within or across sites may also depend on the EBI under implementation.

It is important to acknowledge that there are determinants that cannot be sufficiently addressed through implementation strategies within project initiatives such as Neo-MILK (e.g. external drivers). For a comprehensive understanding of the impact, however, it is important to know what these influencing factors are. Through the application of CFIR 2.0 [45], we were able to categorise the identified determinants into four domains: inner setting, outer setting, individual, and implementation process. Determinants within the outer setting domain, in particular, pose challenges. Such factors often include political influences and broader macro-structural conditions. While specific strategies can be developed to address these factors, they are often beyond the scope of local implementation teams [48, 49]. It is therefore important to recognise the limitations of attempting to address these external conditions, as this recognition allows for a more nuanced understanding of how these factors may affect the success or failure of an implementation intervention.

### Implications for research and practice

The findings of this study have several implications for implementation research and practice. We present these as potential applications that warrant further investigation rather than as empirically demonstrated conclusions.

The SSM-MMCS methodology as described here could serve as a structured approach for the early stages of implementation tailoring, specifically the identification and analysis of implementation determinants [2, 3]. By generating outputs that differentiate between homogeneous and heterogeneous determinant patterns, the approach may inform decisions about whether implementation strategies should be designed at a cross-site level or adapted to local conditions. We note, however, that the present study focused on describing and demonstrating the methodology; the subsequent steps of strategy selection and adaptation were outside the scope of this paper, on which more research is needed [1, 4, 50, 51].

The iterative application of SSM across multiple evaluation timepoints, as designed in the Neo-MILK formative evaluation, holds potential for monitoring shifts in implementation determinants over time. During the ongoing implementation process, the maps initially created can be constantly compared with the evolving implementation context, allowing for the detection of shifts in barriers and their related impact. Such ongoing comparison may provide valuable insights that can inform the refinement of already deployed implementation strategies or the definition of new ones. SSM may also serve as a valuable tool in the event that the first SSM assessment is conducted after the deployment of initial strategies (based on existing literature, experience, or non-context-specific surveys), as the insights gained can enhance the refinement or redefinition of these strategies. Although this longitudinal application is not demonstrated in the present paper (which reports only the first loop), it represents a promising direction for ongoing context monitoring in implementation research.

Furthermore, there are tools that can be employed in conjunction with the findings of an SSM-MMCS analysis in the further tailoring process, including the ERIC matching tool [18] and the StrategEase tool [52]. Such tools can play a pivotal role in ensuring that implementation strategies remain aligned with evolving contextual challenges and interest-holder needs. Crucially, the involvement of relevant interest-holders is essential [1]. It may be beneficial to engage a more diverse set of interest-holders, including specific professional groups pertinent to the context (e.g., in the context of our study, lactation consultants, physicians, nurses, and management) and the target population of the intervention (e.g., mothers and parents of VLBW infants).

### Limitations

This work has limitations that should be acknowledged. While SSM has typically been used as a self-reporting tool, we adapted it to analyse and visualise the content of key person interviews we conducted. The use of interviews introduces the possibility of social desirability bias in responses, which may influence the accuracy of the data collected. We did not specifically gather feedback on whether the results and findings generated by the SSM approach were perceived as helpful by the local implementation teams and the *support system* (project coordination team and intervention development team). It is therefore not possible to provide a definitive assessment of the practical utility of SSM in guiding the implementation process from the perspective of those directly involved. Moreover, we were not able to evaluate whether SSM is appropriate, acceptable, or feasible when applied by local implementation teams. However, other publications on SSM indicate that it is indeed appropriate for such use [23–25]. As SSM was not employed at the outset of the project, a comparison of maps in time cannot be presented, which would have provided insights into how implementation determinants evolved over time. It is also important to note that the responsibility for selection and design of implementation strategies based on the SSM results did not fall within the remit of our evaluation team. We were therefore only able to describe and demonstrate a methodology for the identification and analysis of implementation determinants (steps 1–6), rather than demonstrating the subsequent steps of strategy selection and refined implementation strategies. Although we have outlined potential applications, further research is needed to fully explore the role of SSM in the design and refinement of tailored implementation strategies. Finally, the application reported in this paper involved five NICUs and 10 interviews. While this was appropriate for the methodological demonstration, the transferability of the approach to other settings and larger samples requires further investigation.

## Conclusions

In this study, we described and illustrated the combined application of SSM and the MMCS approach as a structured six-step methodology for identifying and analysing implementation determinants within and across implementation sites. Applied within the FE of a multi-site NICU effectiveness-implementation study, the methodology generated structured outputs that distinguish between homogeneous and heterogeneous determinant patterns, a distinction with potential relevance for differentiating between cross-site and site-specific implementation strategies. The six steps outlined in this paper may offer a replicable approach for gaining a comprehensive understanding of local implementation contexts by systematically considering the responsibilities, needs, resources, and wishes of individual interest-holders. Where determinants show homogeneous genesis and impact across sites, the findings may inform the development of cross-site implementation strategies, enabling the efficient bundling of implementation capacities. Where determinants are heterogeneously perceived, site-specific assessment and locally adapted strategies appear warranted. Whilst these implications are derived from a single application, they suggest that embedding SSM within the early stages of both initial and ongoing implementation tailoring may enhance the contextual responsiveness of strategy design. Nevertheless, further research is needed to evaluate the use of SSM across multiple evaluation timepoints and its integration into the ongoing tailoring of implementation strategies.

## Supplementary Information

Below is the link to the electronic supplementary material.

Supplementary Material 1

Supplementary Material 2

Supplementary Material 3

Supplementary Material 4

Supplementary Material 5

Supplementary Material 6

Supplementary Material 7

## Data Availability

The datasets generated and/or analysed during the current study are not publicly available, as this would likely compromise the anonymity of the participants, particularly to other parties involved in the Neo-MILK project. Some descriptive data may be made available by the corresponding author upon reasonable request.

## Abbreviations

CFIR: Consolidated framework for implementation research
COREQ: Consolidated criteria for reporting qualitative research
c-RCT: cluster-randomised controlled trial
EBI: Evidence-based intervention
FE: Formative evaluation
HDM: Human donor milk
HDMB: Human donor milk bank
IFS: Interactive systems framework for dissemination and implementation
MMCS: Matrixed multiple case study
MOM: Mother’s own milk
NICU: Neonatal intensive care unit
pCAT: pragmatic context assessment tool
SOP: Standard operating procedure
SSM: System support mapping
TIDieR: Template for intervention description and replication
TRAPD: Translation, review, adjudication, pretest, documentation
VLBW: Very low birth weight
