# Supplementary material for: Applying system support mapping combined with the matrixed multiple case study approach to identify and analyse implementation determinants: lessons from a multi-site implementation study in German neonatal intensive care units
Source: BMC Health Serv Res. 2026 Sep 5;26:1238. doi: 10.1186/s12913-026-15507-w (PMC13551688; doi:10.1186/s12913-026-15507-w)
Supplement: Supplementary file 1 [file 12913_2026_15507_MOESM1_ESM.pdf]

Additional file 1: Description of System Support Mapping (SSM)

Based on the role of an individual, which is located in the centre of the ring-shaped structure of a System Support Map (see Figure 1), each individual captures its role's responsibilities and key needs for completing each responsibility in the next outer rings. Continuing outwards, the resources that meet these needs and the three most important wishes for how an individual could be supported in their work are recorded. After filling out each ring, responses from one ring to the next are linked. As System Thinking emphasises the importance of examining the relationships between actions and their context, connecting responses is a crucial step within the SSM process, what provides rich insights and promotes a comprehensive understanding of the system as a whole. Completing a map can be supported by an online program, which guides participants through the creating process of an SSM using customised questions and prompts to generate responses for each SSM element (ring) and link them. The approach has traditionally been used as a self-assessment tool to capture and analyse the roles and responsibilities of healthcare professionals related to a defined intervention aiming at improving maternal and child health. It has been used to enhance team collaboration, breaking down barriers to collaboration, and developing strategies to improve work efficiency and collaboration [33].

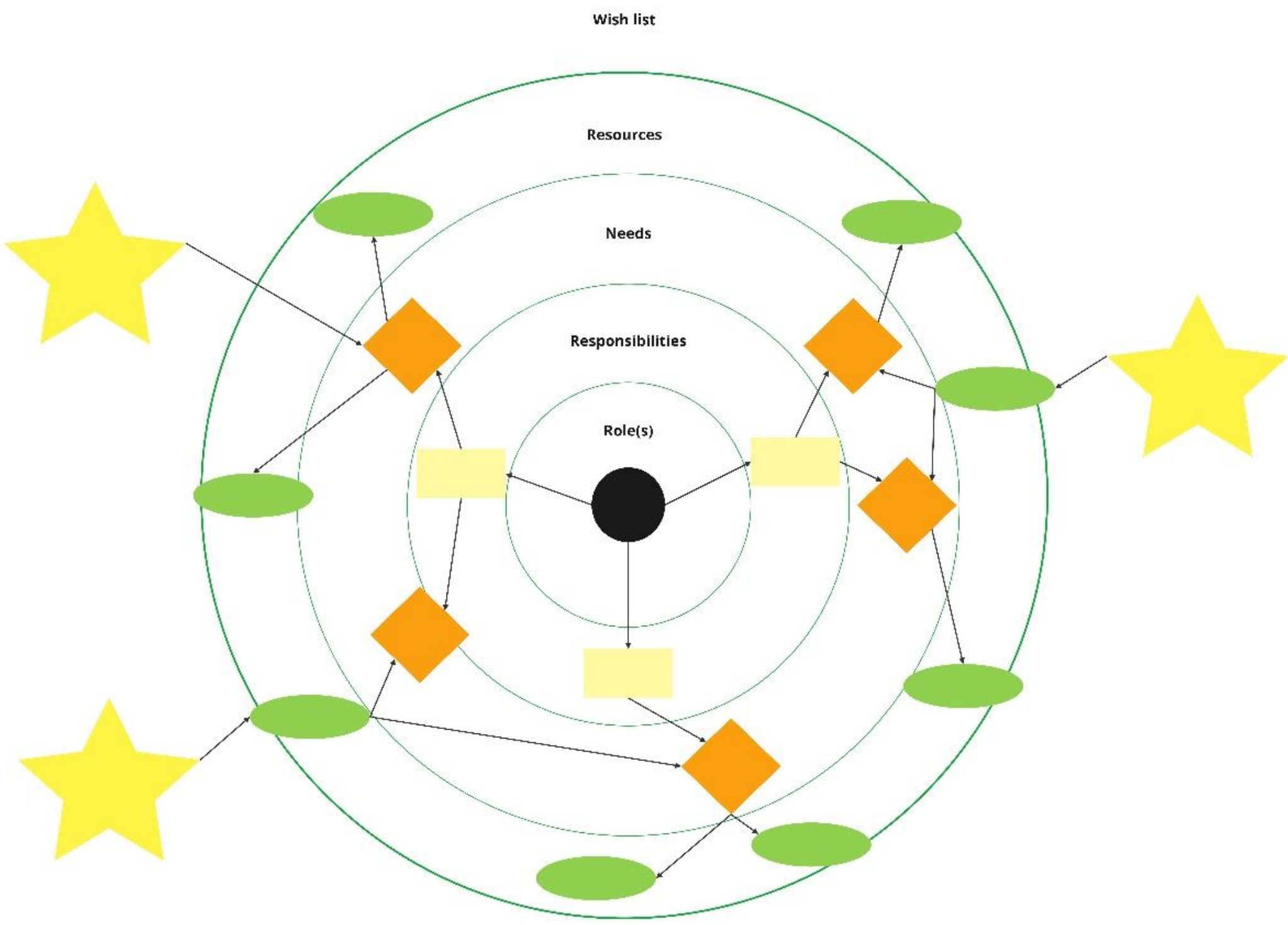

Figure 1: Example System Support Map without content to illustrate its visualisation in accordance with Calancie et al. [33]

SSM elements are displayed as follows: role(s) in a black circle, responsibilities in yellow rectangles, needs in orange diamonds, resources in green ovals, and wishes in gold stars. The arrows indicate that the arrow source is connected to the element the arrow points to (e.g., a role is related to a particular responsibility, or a resource helps with a specific need).
