# Supplementary material for: Applying system support mapping combined with the matrixed multiple case study approach to identify and analyse implementation determinants: lessons from a multi-site implementation study in German neonatal intensive care units
Source: BMC Health Serv Res. 2026 Sep 5;26:1238. doi: 10.1186/s12913-026-15507-w (PMC13551688; doi:10.1186/s12913-026-15507-w)
Supplement: Supplementary file 2 [file 12913_2026_15507_MOESM2_ESM.pdf]

Additional file 2: Consolidated Criteria for Reporting Qualitative Studies (COREQ): 32-item checklist

No	Item	Guide questions/description	Manuscript page
Domain 1: Research team and reflexivity			
**Personal Characteristics**			
**1.**	Interviewer/facilitator	Which author/s conducted the interview or focus group?	11
**2.**	Credentials	What were the researcher's credentials? *E.g. PhD, MD*	11
**3.**	Occupation	What was their occupation at the time of the study?	11, 12
**4.**	Gender	Was the researcher male or female?	11
**5.**	Experience and training	What experience or training did the researcher have?	11,12
**Relationship with participants**			
**6.**	Relationship established	Was a relationship established prior to study commencement?	n/a
**7.**	Participant knowledge of the interviewer	What did the participants know about the researcher? e.*g. personal goals, reasons for doing the research*	n/a
**8.**	Interviewer characteristics	What characteristics were reported about the interviewer/facilitator? e.g. *Bias, assumptions, reasons and interests in the research topic*	n/a
Domain 2: study design			
**Theoretical framework**			
**9.**	Methodological orientation and Theory	What methodological orientation was stated to underpin the study? *e.g. grounded theory, discourse analysis, ethnography, phenomenology, content analysis*	12
**Participant selection**			
**10.**	Sampling	How were participants selected? *e.g. purposive, convenience, consecutive, snowball*	n/a
**11.**	Method of approach	How were participants approached? e.*g. face-to-face, telephone, mail, email*	11
**12.**	Sample size	How many participants were in the study?	11, 16
**13.**	Non-participation	How many people refused to participate or dropped out? Reasons?	20, 21
**Setting**			
**14.**	Setting of data collection	Where was the data collected? e.*g. home, clinic, workplace*	11
**15.**	Presence of non-participants	Was anyone else present besides the participants and researchers?	11
**16.**	Description of sample	What are the important characteristics of the sample? *e.g. demographic data, date*	16
**Data collection**			
**17.**	Interview guide	Were questions, prompts, guides provided by the authors? Was it pilot tested?	11

**18.**	Repeat interviews	Were repeat interviews carried out? If yes, how many?	n/a
**19.**	Audio/visual recording	Did the research use audio or visual recording to collect the data?	11, 12
**20.**	Field notes	Were field notes made during and/or after the interview or focus group?	n/a
**21.**	Duration	What was the duration of the interviews or focus group?	11
**22.**	Data saturation	Was data saturation discussed?	n/a
**23.**	Transcripts returned	Were transcripts returned to participants for comment and/or correction?	n/a
Domain 3: analysis and findings			
**Data analysis**			
**24.**	Number of data coders	How many data coders coded the data?	12
**25.**	Description of the coding tree	Did authors provide a description of the coding tree?	12, 13
**26.**	Derivation of themes	Were themes identified in advance or derived from the data?	12, 13
**27.**	Software	What software, if applicable, was used to manage the data?	12
**28.**	Participant checking	Did participants provide feedback on the findings?	27
**Reporting**			
**29.**	Quotations presented	Were participant quotations presented to illustrate the themes / findings? Was each quotation identified? e.*g. participant number*	n/a
**30.**	Data and findings consistent	Was there consistency between the data presented and the findings?	n/a
**31.**	Clarity of major themes	Were major themes clearly presented in the findings?	16-25
**31.**	Clarity of minor themes	Is there a description of diverse cases or discussion of minor themes?	25-26
