# Supplementary material for: Applying system support mapping combined with the matrixed multiple case study approach to identify and analyse implementation determinants: lessons from a multi-site implementation study in German neonatal intensive care units
Source: BMC Health Serv Res. 2026 Sep 5;26:1238. doi: 10.1186/s12913-026-15507-w (PMC13551688; doi:10.1186/s12913-026-15507-w)
Supplement: Supplementary file 3 [file 12913_2026_15507_MOESM3_ESM.pdf]

Additional file 3: Interview guide

**Introduction**	<u>Introduction of the interviewer</u><br><br>Thank you very much for taking the time for this interview and for agreeing to take part. Before we start the interview, I would like to explain to you briefly what this interview is about and what its purpose is.<br><br>Before your NICU moves from the control phase to the intervention phase, we would like to explore, with your help, the planned implementation processes in your NICU. Specifically, we would like to explore the daily care you provide without the influence of the intervention and the processes you use to prepare for implementation. In particular, we would like to explore your motivations for participating in the Neo-MILK research project, your current work routines, your expectations and attitudes towards implementing the intervention into your daily work routines, and identify potential barriers and facilitators to implementation.<br><br>We have estimated the interview to take approximately 45-60 minutes, but this may vary depending on how the interview goes. If you need a break during the interview, please let me know. If you agree, I would like to record the interview using the recording function (zoom) so as not to interrupt the flow of the interview and to make it easier for us to analyse later. The interview will be kept confidential and the evaluation will be anonymous. The recording will be deleted after the evaluation and will not be passed on to third parties. You have the option at any time to request that parts of the interview not be used for analysis.<br><br>Please do not hesitate to ask any questions you may have prior to the commencement of the interview. Once you are satisfied that you are adequately prepared, we can proceed.	Introduction<br><br>Aim<br><br>Procedure<br><br>Data protection

No.	Key question / main question	Detailed questions / follow-up questions	SSM element(s) primarily addressed	Rationale for SSM-mapping
**1**	To begin with, I would like to know how you decided to take part in the Neo-MILK project?	What need(s) have been identified for the target population(s) and what are the causes of these needs?<br><br>What could change as a result of the implementation of the intervention components (breastfeeding promotion concept and human donor milk bank) for this population(s)?	Not element-specific	Not applicable
**2**	I am interested in the health care process for mothers and their children in your NICU.<br><br>Could you please describe an example of the health care routine from the time a mother is admitted to your clinic?	Please tell me about a situation in which you perceived the health care process as particularly good.<br><br>Please describe a situation in which you experienced a less than optimal health care process.	Not element-specific	Not applicable
**3**	What expectations do you have of the intervention in terms of the health care process for mothers and their children?<br><br>Please tell me about your expectations.	What might change in the care process for mothers and children?<br><br>What advantages compared to your previous practice/past changes might result?<br><br>How will you determine whether the project was successful for your organisation?	Wishes, Needs	Questions about expectations regarding the intervention and anticipated changes reflect what key persons would ideally like to see in place (wishes) and which conditions they consider necessary (needs)

**4**	I am particularly interested in the plans regarding the implementation of the lactation and breastfeeding support programme and the human donor milk bank.<br><br>Please explain how you will implement the intervention components with the resources available to your organisation.	Are you planning to implement all or only some of the intervention components? If partially, why and which components?<br><br>Please describe your personal ideal of the conditions and available resources that you consider to be a prerequisite for optimal implementation.<br><br>What factors could have a facilitating impact on implementation?<br><br>What factors could emerge as challenges?	Resources, Responsibilities, Needs	The central SSM-informed question: covers available resources for implementation, planned actions and tasks (responsibilities), and perceived prerequisites and challenges (needs)
**5**	To what extent are you supported by the project coordination team during the implementation, but also during the realisation of the intervention components in your daily routine?	What other support would you like to receive?	Resources, Wishes	Support provided by the project coordination team represents an external resource; desired additional support reflects what key persons would wish to have available (wishes)
**6**	Finally, I would like to know what role you personally see yourself playing in the implementation and realisation of the intervention?	Please describe your responsibilities and the associated tasks.<br><br>What other responsibilities are there and what tasks do they take on?	Roles, Responsibilities	Direct question about the key person's own role and the tasks and responsibilities associated with it, as well as those held by others involved in implementation
**7**	Is there anything else you would like to add?		Not element-specific	Not applicable

Note: The interview guideline served both the process evaluation of the Neo-MILK c-RCT and the SSM-based analysis presented in this paper. Questions were phrased in the everyday professional language of the interviewees rather than in SSM terminology; the SSM elements were applied as analytical categories during coding (see Methods, step 2). The annotations above therefore indicate which element(s) a question was primarily intended to inform and do not imply a one-to-one correspondence between question wording and element label.
