# Supplementary material for: Applying system support mapping combined with the matrixed multiple case study approach to identify and analyse implementation determinants: lessons from a multi-site implementation study in German neonatal intensive care units
Source: BMC Health Serv Res. 2026 Sep 5;26:1238. doi: 10.1186/s12913-026-15507-w (PMC13551688; doi:10.1186/s12913-026-15507-w)

Additional file 4: Reported impact and mode of action of each resources category across all NICUs

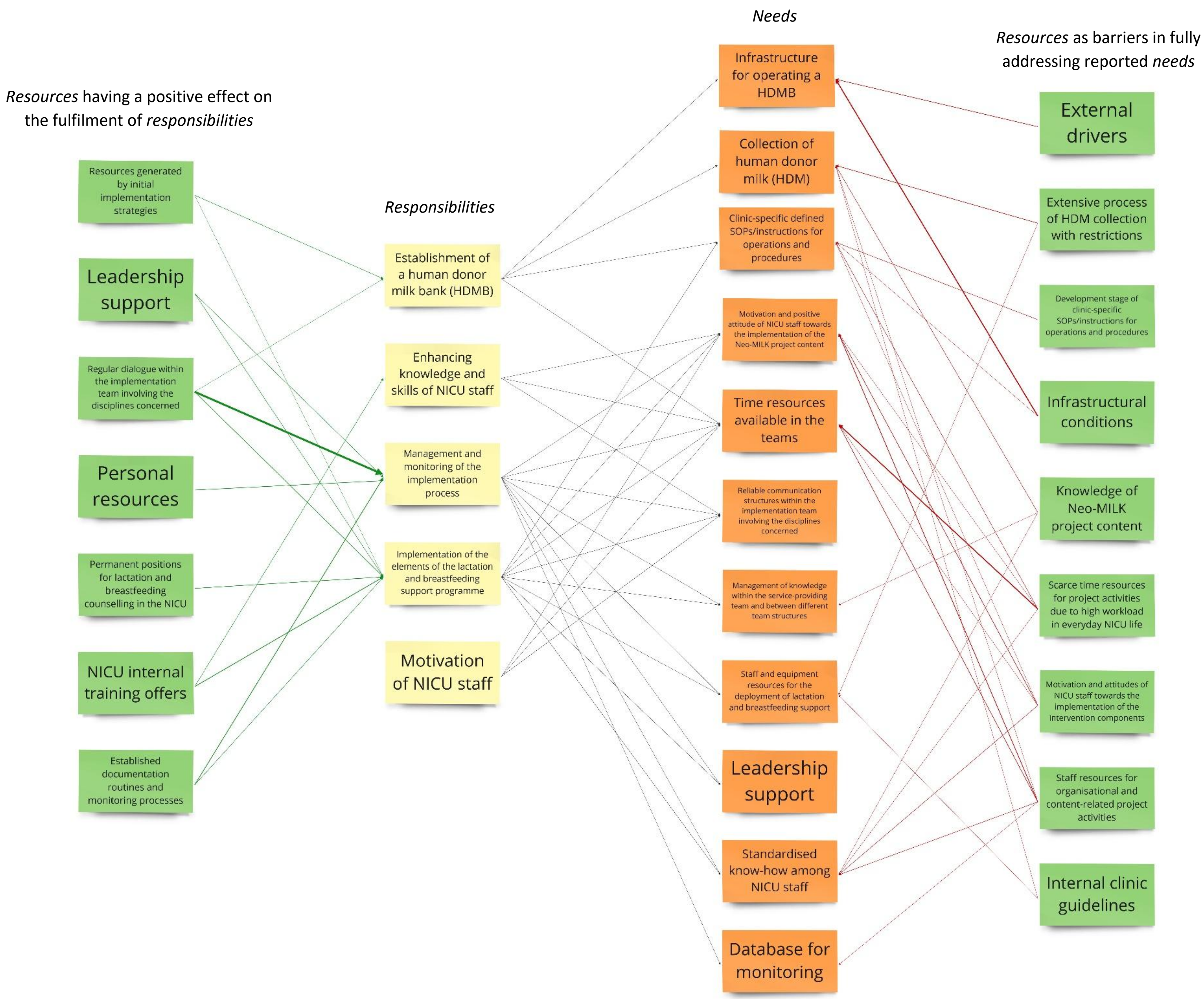

Supplement: Supplementary file 4 [file 12913_2026_15507_MOESM4_ESM.pdf]
