# Supplementary material for: Applying system support mapping combined with the matrixed multiple case study approach to identify and analyse implementation determinants: lessons from a multi-site implementation study in German neonatal intensive care units
Source: BMC Health Serv Res. 2026 Sep 5;26:1238. doi: 10.1186/s12913-026-15507-w (PMC13551688; doi:10.1186/s12913-026-15507-w)
Supplement: Supplementary file 5 [file 12913_2026_15507_MOESM5_ESM.pdf]

Supplementary Table 1: Components of the Neo-MILK intervention in accordance with TIDieR [31]

Brief name	Why?	What?	Who provided?	How?	Where?	When and how much?
**Prepartum consultation on lactation and breastfeeding between mothers, i.e. parents, and staff member of the NICU**	The prepartum consultation aims to educate, raise awareness and increase knowledge in the area of lactation and breastfeeding in mothers/parents.	The prepartum consultation provides information on significant topics such as (1) bonding and first contact of the mother, i.e. parents with the VLBW infant, (2) the impact of breastfeeding, colostrum and breast milk, (2) lactation in prematurity, (3) expression of breast milk, (4) hygienic handling of expressed breast milk as well as (5) information on NICU accessibility and (6) contact and information for the respective clinic.	The prepartum consultation is provided by a neonatologist working on the respective NICU.	The prepartum consultation should be delivered to mothers, i.e. parents, individually.	The consultations take place in a separate room of the clinic (e.g. patient room, physician office).	The one-time consultation is intended to take place prepartum. In case of an unexpected birth, information should be provided to mothers/parents after the VLBW infant has been admitted to the NICU.

Brief name	Why?	What?	Who provided?	How?	Where?	When and how much?
**Question prompt sheet (QPS) for the preparation of prepartum consultation on lactation and breastfeeding between mothers, i.e. parents, and staff member of the neonatal intensive care unit (NICU)**	The QPS offers example questions on significant topics such as lactation after preterm birth, bonding, and hygienic handling of expressed breast milk, in line with the information provided during the prepartum consultation. The QPS functions as a preparation aid for mothers, or parents, for the prepartum consultation, and a reference resource for later questions or for refreshing previously acquired knowledge from the consultation.	The QPS is provided as a printed booklet. It provides sample questions on the topics addressed in the prepartum consultation. Also, there is additional space for questions and further notes. Moreover, simplified definitions i.e., explanations of technical terms relevant to prepartum counselling are included.<br><br>To prepare for the consultation or follow-up consultations after delivery, mothers, i.e. parents read through the booklet and may add questions. The QPS is then taken to consultations with HCPs to support communication and collect information provided during the consultation using pages provided for notes. It can be reviewed at any time, both between and after consultations.	The QPS is provided by healthcare providers (HCPs) working on the respective NICU (trained nurses, midwives and physicians).	The QPS should be delivered to mothers, i.e. parents, individually. Mothers, i.e. parents administer the QPS individually.	The QPS should be delivered to mothers, i.e. parents, in a separate room of the clinic (e.g. patient room, physician office).	The QPS is intended for a one-time administration before the first (prepartum) consultation.<br><br>The same QPS can however be used multiple times by mothers, i.e. parents for follow-up consultations or for self-study to refresh their knowledge.

Brief name	Why?	What?	Who provided?	How?	Where?	When and how much?
**Flyers on lactation, breastfeeding, and human donor milk**	The provision of flyers aims to educate, raise awareness and increase knowledge in the area of lactation, breastfeeding and human donor milk in mothers/parents.	The printed flyers are written in German and provide information on the following nine topics: (1) Neo-MILK app in general, (2) Neo-MILK films within the app, (3) information for fathers and partners, (4) manual let-down, (5) hygienic handling of expressed breast milk, (6) physical contact and bonding/ kangarooing, (7) strategies for solving lactation or breastfeeding related problems, (8) expression of breast milk, and (9) human donor milk.<br>In addition, four flyers with the topics 4, 5, 7, 8) are available in Russian, Arabic, Turkish, French, and English.	Flyers are provided by HCPs working on the respective NICU.	Flyers should be delivered to mothers, i.e. parents, individually by handing them over. Additionally, the flyers should be displayed by HCPs on the NICU (e.g., in pumping rooms, ward rooms).	Flyers should be handed over and be displayed on the NICU.	Flyers are intended for a one-time administration to mothers/parents but they should be refilled when not anymore available on the NICU.<br><br>The flyers can however be used multiple times by mothers, i.e. parents for self-study to refresh their knowledge.

Brief name	Why?	What?	Who provided?	How?	Where?	When and how much?
**Posters on lactation, breastfeeding, feeding VLBW infants with mother's own milk (MOM), and information on the specificity of the respective NICU being a trial site**	The purpose of displaying posters is to educate, raise awareness and increase knowledge in the area of lactation, breastfeeding, and feeding VLBW infants with MOM in mothers/parents. Additionally, the posters should provide information on the specificity of the respective NICU being a trial site.	The printed posters are written in German, available in different sizes (DIN-A4 and DIN-A3), and provide information on the following topics: (1) feeding VLBW infants with MOM, (2) breastfeeding positions, (3) effective expression of breast milk, (4) bonding/ kangarooing, (5) DOs on the NICU, (6) Neo-MILK app, (7) specificity of the respective NICU being a trial site, (8) body care, (9) breast milk, (10) breast milk flow chart	Posters are provided by HCPs working on the respective NICU.	Posters should be made accessible to everyone on the NICU, e.g. by hanging them up.	Posters should be opened in the NICU, e.g. in the NICU corridors as well as in the pumping and breastfeeding rooms.	Posters are intended for a one-time administration, but can be extended or repositioned at any time.<br><br>The posters can however be used multiple times by mothers, i.e. parents for self-study to refresh their knowledge.

Brief name	Why?	What?	Who provided?	How?	Where?	When and how much?
**App-based support on lactation and breastfeeding and to reflect on breast milk production**	The provision of app-based support aims to educate, raise awareness and increase knowledge in the area of lactation, breastfeeding and is intended to help mothers reflect on their breast milk production	The app can only be accessed online via **https://app.neo-milk.de/#/welcome**. It is not possible to install the app on a mobile device.<br><br>The app contains information and instructions on the topics of skin-to-skin contact, breast milk, breast milk expression and everyday tips. Informational content in film format is also available. Pumping reminders can be set up to document the milk quantity and pumping/breastfeeding times as well as the pumping/breastfeeding duration with the help of a stopwatch. This can be used to create a personalised milk chart. The app also offers the option of creating diary entries, notes and a picture gallery. Information on the study and the intervention is also provided.	Information on the app and the access to it are displayed on printed flyers and posters, which are provided by HCPs working in the respective NICU.	The app can be accesses and used online. There is a QR code on the flyer containing information about the films to make the website easily accessible.<br><br>Registration with an email address is required to use all features.	The app can be accessed and used online from home, on the move or from the NICU.	The access and use of the app are voluntary. There is no specified time frame for using the app.

Brief name	Why?	What?	Who provided?	How?	Where?	When and how much?
**Human Donor Milk Bank (HDMB)**	The establishment or expansion of HDMB will provide the opportunity to access HDM from the first day of life and, if needed, until discharge.	Every VLBW infant, who is in need for HDM will have access until discharge. Furthermore, mothers of VLBW infants and who are potential donors have the opportunity to donate.	The need for HDM and the potential to donate are assessed by HCPs working in the NICU.	Procedures depend on NICU-specific HDMB concepts and have been developed by the NICUs themselves.	The HDMB can be accessed in the NICU.	Access is initiated when an infant is in need or a mother is medically eligible and willing to donate. The infant can receive HDM until discharge.
