# Supplementary material for: Applying system support mapping combined with the matrixed multiple case study approach to identify and analyse implementation determinants: lessons from a multi-site implementation study in German neonatal intensive care units
Source: BMC Health Serv Res. 2026 Sep 5;26:1238. doi: 10.1186/s12913-026-15507-w (PMC13551688; doi:10.1186/s12913-026-15507-w)
Supplement: Supplementary file 6 [file 12913_2026_15507_MOESM6_ESM.pdf]

Supplementary Table 2: Specification of the implementation strategies of Neo-MILK as suggested by Proctor et al. [33]

	Training and certification for NICU staff	Provision of manuals on lactation and breastfeeding	Provision of information material for NICU staff	Provision of checklist for prepartal consultation	Increasing commitment through nudging	Provision of SOPs for setting up the HDMB	Financial resource
**Actor(s)**	Project coordinator	Project coordinator	Project coordinator	Project coordinator	Project coordinator	Project coordinator	Statutory health insurances Funder: G-BA
**Actions(s)**	Online-training on the lactation and breastfeeding support intervention	Support through guidelines (instructions for action)	Support through posters on lactation and breastfeeding, and fact sheets	Support through guidelines (conversation guide)	Nudging (e.g. obtaining commitment in written form or public presentation through posters)	Support through procedural policies	Neo-MILK intervention is part of a funded project of the G-BA, the NICUs get financial support (NICUs receive equipment for setting up the human donor milk bank and funds to hire staff)
**Target(s) of action**	NICU staff and other affiliated departments (if desired)	NICU staff	NICU staff	NICU staff	NICU staff	NICU	NICU
**Temporality**	Beginning of intervention phase in respective NICU	Throughout intervention phase in respective NICU with start at the beginning of intervention phase	Throughout intervention phase in respective NICU with start at the beginning of intervention phase	Throughout intervention phase in respective NICU with start at the beginning of intervention phase	Throughout intervention phase in respective NICU with start at the beginning of intervention phase	Throughout intervention phase in respective NICU with start at the beginning of intervention phase	HDMB equipment: intervention phase, Infrastructural payments: Beginning of the control phase
**Dose**	2 h training (crash course), 8 h training (long version)	Initial setup of material, need-based restocking	Initial setup of material, need-based restocking	Initial setup of material, need-based restocking	Initial setup of material, need-based restocking	Initial setup of material, need based restocking	One-time delivery of the HDMB equipment, yearly payments for infrastructural support

**Implementation outcome(s) affected**	Adoption, Implementation (Fidelity)	Adoption, Implementation (Fidelity), Sustainability Reaction/Response (organisation)	Adoption, Implementation (Fidelity)	Implementation (Fidelity)	Adoption, Sustainability	Implementation (Penetration), Sustainability	Adoption, Implementation (Costs)
**Justification**	Educational training is a necessary but insufficient strategy for behaviour change. Building knowledge is important for laying the foundation for the actual application of the intervention components.	Educational materials are important to support the NICU staff learning how to deliver the intervention, which may influence the uptake, delivery and maintenance of the intervention. Distributing educational materials promotes to increase self-efficacy and a positive attitude towards the intervention and supports establishing a pro-implementation attitude.	Information materials are useful for summarizing knowledge for NICU staff about the intended mode of delivering the intervention.	Check lists are important to support the NICU staff in delivering the intervention. It provides guidance and a rough list of essential steps to achieve the function of a specific intervention component.	Nudging could improve the NICU staff's commitment to be involved in delivering the intervention and engaging in its sustained use.	SOPs and other procedural policies support the NICU staff setting up and embed the intervention into routine practice.	Implementing a new model of care is a highly complex process of change in the routine care of the clinics, which are subject to financial pressure anyway. Financial support is necessary to support the adoption of new interventions like Neo-MILK, to optimize the implementation and prepare its maintenance.
