# Supplementary material for: Applying system support mapping combined with the matrixed multiple case study approach to identify and analyse implementation determinants: lessons from a multi-site implementation study in German neonatal intensive care units
Source: BMC Health Serv Res. 2026 Sep 5;26:1238. doi: 10.1186/s12913-026-15507-w (PMC13551688; doi:10.1186/s12913-026-15507-w)
Supplement: Supplementary file 7 [file 12913_2026_15507_MOESM7_ESM.pdf]

Supplementary Table 3: Categories derived from inductively developed codes for each SSM element using axial coding

SSM element	Category derived from inductively developed codes for each SSM element using axial coding	Inductively developed code within each SSM element
**Role(s)**	**Local project lead**	Local project lead
	**Coordinator**	Coordinator
	**Motivator**	Motivator
	**Supervisory authority**	Supervisory authority
	**Operational implementation instance**	Operational implementation instance

SSM element	Category derived from inductively developed codes for each SSM element using axial coding	Inductively developed code within each SSM element
**Responsibilities**	**Establishment of a human donor milk bank (HDMB)**	Overview of HDM available following the launch of a HDMB
		Donor acquisition
		Development of instructions for operations and procedures for HDMB operation
	**Enhancing knowledge and skills of NICU staff**	Training of NICU staff
		Raising awareness for the significance of breast milk nutrition
	**Motivation of NICU staff**	Motivation of NICU staff
	**Management and monitoring of the implementation process**	Monitoring of the implementation's success
		Addressing implementation / realisation issues
		Communication between medical professionals, nursing staff and lactation and breastfeeding counsellors
	**Implementation of the elements of the lactation and breastfeeding support programme**	Conducting prepartum information sessions
		Support for mothers during pumping/breastfeeding
		Ensuring comprehensive lactation and breastfeeding counselling

SSM element	Category derived from inductively developed codes for each SSM element using axial coding	Inductively developed code within each SSM element
**Needs**	**Leadership support**	Leadership support
	**Motivation and positive attitude of NICU staff towards the implementation of the Neo-MILK project content**	Motivated staff
		Sense of responsibility
		Openness to change among NICU staff
	**Standardised know-how among NICU staff**	Trained consultation staff
		Trained support staff
		Regular training sessions
	**Reliable communication structures within the implementation team involving the disciplines concerned**	Regular coordination within the project team
		Regular interdisciplinary dialogue
		Coordination of hygiene when handling pump sets
	**Database for monitoring**	Identification of implementation/realisation problems
		Identification of need for further action
		Data collection
	**Staff and equipment resources for the deployment of lactation and breastfeeding support**	Staff available for lactation and breastfeeding counselling
		Staff for placing the Neo-MILK materials
		Options for placement
	**Time resources available in the teams**	Leave of absence for 2.5 hours of training
		Free valences
	**Management of knowledge within the service-providing team and between different team structures**	Access to NICU staff
		Knowledge about pumping/breastfeeding problems of mothers
		Information about imminent preterm birth
	**Clinic-specific defined SOPs/instructions for operations and procedures**	Internal guidelines
		Defined instructions for operations and procedures
		Hygiene concept
	**Infrastructure for operating a HDMB**	(Digital) infrastructure
		Additional milk testing staff
		Financial resources for milk testing
	**Collection of human donor milk (HDM)**	Available HDM
		Obtaining human donor milk
		Donors

SSM element	Category derived from inductively developed codes for each SSM element using axial coding	Inductively developed code within each SSM element
Resources	**NICU internal training offers**	Advocacy for training to be credited as working time
		Internal training on lactation and breastfeeding
		Internal training centre (“parent school”)
	**Regular dialogue within the implementation team involving the disciplines concerned**	Sovereignty of nursing staff in operational decision-making
		Regular interdisciplinary dialogue
		Regular project team meetings
	**Leadership support**	Support from the Executive Board
		Support from medical director
		Past critical attitude of the medical director towards breast milk
	**Staff resources for organisational and content-related project activities**	Staff available for training NICU staff
		Staff available for lactation and breastfeeding counselling
		Shortage of nursing staff
	**Infrastructural conditions**	Lack of devices and software
		Digital infrastructure
		Interim premises
	**External drivers**	Policymakers
		Secured funding
		Uncertain funding sources
	**Scarce time resources for project activities due to high workload in everyday NICU life**	Scarce time resources
		High workload in everyday NICU life
	**Personal resources**	Vacation, parental leave
		ICU experience
		Management position
	**Motivation and attitudes of NICU staff towards the implementation of the intervention components**	Varying levels of motivation among NICU staff
		Ingrained processes and attitudes of NICU staff
		Poor learning mentality
	**Extensive process of HDM collection with restrictions**	Long lead times for bacteriological testing
		Limited storage life of the milk
		Mothers' own initiative
	**Development stage of clinic-specific SOPs/instructions for operations and procedures**	Undefined SOPs
		Undefined instructions for operations and procedures
		Adapted HACCP concept
	**Resources generated by initial implementation strategies**	Legal reports
		Hygienic and infectiology standard
		Neo-MILK synergies
	**Knowledge of Neo-MILK project content**	Widespread knowledge of basics on breastfeeding among NICU staff
		Low awareness of early lactation

		Insufficient information flow
	**Permanent positions for lactation and breastfeeding counselling in the NICU**	Commitment for leave of absence for IBCLC training
		Limited leave of absence for lactation and breastfeeding counselling
		Subsidiary lactation and breastfeeding counselling from different ward
	**Established documentation routines and monitoring processes**	Project-independent monitoring
		Documentation of pumping/breastfeeding experience in electronic patient file
		Information list for donation acceptance
	**Internal clinic guidelines**	Deviating hygiene measures when handling the pumping set
		Objections of hygiene regarding raw milk
		Laboratory not certified for food testing

SSM element	Category derived from inductively developed codes for each SSM element using axial coding	Inductively developed code within each SSM element
**Wishes**	**Support from policymakers, regulatory authorities and professional associations**	Recognition by professional associations of the special nature of breast milk
		Financial support for milk testing
		Support with regulatory processing for the establishment of a HDMB
	**Enhanced collaboration through improved awareness of the importance of breast milk feeding**	Opportunities for dialogue with other intervention clinics
		Strengthened team mentality
		Improved cooperation with the delivery room for early lactation
	**Exclusive feeding with breast milk, alternatively with HDM**	Permanent withdrawal from commercial, pasteurised milk
		Exclusive feeding with human milk
		Optimisation of lactation and breastfeeding support in a way that eliminates the necessity of HDM
	**Increase in staff with permanent responsibilities**	Continuous specialist responsibility for HDMB
		Additional leave of absence for lactation and breastfeeding counselling
		Continued medical responsibility
	**Standardised knowledge of pumping, breastfeeding and HDM among NICU staff to provide comprehensive advice to mothers/parents**	Comprehensive, standardised knowledge of the basics of pumping/breastfeeding
		Elimination of fears of inability to respond to questions raised by mothers
		Increased awareness for HDMB in own clinic
	**Sustainable integration of lactation and breastfeeding-promoting Neo-MILK content into everyday working life**	Early mother-infant contact (directly after birth & before NICU admission)
		Early education of mothers about the importance of breast milk
		Early collection of breast milk
	**Database for monitoring, process optimisation and quality assurance**	Database on feeding raw HDM
		Reduction in the number of infectious diseases
